# Morbidity and Mortality Conferences: A Mini Review and Illustrated Application in Veterinary Medicine

**DOI:** 10.3389/fvets.2018.00043

**Published:** 2018-03-06

**Authors:** Daniel S. J. Pang, Frédérik Rousseau-Blass, Jessica M. Pang

**Affiliations:** ^1^Department of Clinical Sciences, Faculty of Veterinary Medicine, Université de Montréal, Saint-Hyacinthe, QC, Canada

**Keywords:** morbidity and mortality conference, rounds, meeting, errors, adverse events, complications

## Abstract

This mini review presents current knowledge on the role of morbidity and mortality conferences (M&MCs) as a powerful educational tool and driver to improve patient care. Although M&MCs have existed since the early twentieth century, formal evaluation of their impact on education and patient care is relatively recent. Over time, M&MCs have evolved from single discipline discussions with a tendency to focus on individual errors and assign blame, to multidisciplinary, standardized presentations incorporating error analysis techniques, and educational theory. Current evidence shows that M&MCs can provide a valuable educational experience and have the potential to generate measurable improvements in patient care.

## Introduction

Adverse events, defined as a complication caused by medical management and resulting in patient harm, are an unfortunately common occurrence in hospitalized human patients (1–5). While estimates vary depending on the outcome(s) used to define adverse events, up to 4% of all hospitalized human patients will experience a serious negative outcome (prolonged hospital stay, disability at discharge, or death) (1, 2). Importantly, half of all adverse events are preventable (1, 2, 4, 5). As a result, there is room for substantial improvement in patient outcomes through better care (2–5).

The potential for adverse events to drive improved patient care and safety, and serve as a valuable educational resource, has long been recognized in human medicine (6–9). Since their inception in the first half of the twentieth century, morbidity and mortality conferences (M&MCs, also known as M&M rounds and reviews) have been the mechanism to achieve these outcomes, and they are implemented in a wide range of medical specialties, most notably surgery and anesthesia (7, 9). Their use is now mandated by the Accreditation Council for Graduate Medical Education in human medicine and is part of the Practice Standards Scheme of the Royal College of Veterinary Surgeons (10, 11).

Fittingly described as familiar yet lacking a clear definition, at a fundamental level M&MCs comprise of caregivers gathering to review adverse events, with the goals of education and improving care (7). In principle, M&MCs should provide an open forum for the collaborative review of adverse events without fear of retribution or blame. The primary goals should be improving patient care and maximizing the educational benefits of a shared experience (6, 12–14). These can be achieved through: presentation and acknowledgment of error(s) [defined as performance that deviates from the ideal; failure to carry out a planned action as intended (error of execution), or the use of an incorrect or inappropriate plan (error of planning)], analysis and discussion of adverse events and contributing factor(s), identification of means for improvement, dissemination of information, and reinforcement of responsibility to provide best practice standard of care (7, 15–17). In practice, however, M&MCs are often poorly defined in terms of format, goals, and outcomes (7, 18, 19). Learning from errors through reflection and discussion is essential to improve practice, though where this is done ineffectually, or with the emphasis on assigning blame, M&MCs fail to be productive (7, 13, 20).

While there is a large body of literature supporting and advocating the use of M&MCs, their efficacy in terms of measurable outcomes has, until recently, been largely untested. Increasing evidence suggests that a structured, transparent approach to M&MCs results in measurable gains in user satisfaction and participation, education, patient safety, quality of care, and mortality (13, 15, 21–25). This mini review will discuss the demonstrated benefits of M&MCs and available evidence on their optimal format.

## Benefits

### Education

Despite their long history, it has only been relatively recently that prospective trials have been conducted to evaluate the educational contribution of M&MCs (15, 19, 21, 24, 26). These studies have developed and tested structured approaches to M&MCs, encompassing case reporting and selection, analysis of adverse events, presentation, participation, and learning outcomes.

Implementation of a standardized presentation format significantly improved the number of correct responses to multiple choice questions completed at the end of each M&MC and presentation quality (15). The same group had previously developed an M&MC presentation assessment tool using psychometric principles that was feasible (taking <10 min to complete), reliable (high-internal consistency and inter-rater agreement), and valid (construct validity), thus allowing presenters and presentation content to be objectively evaluated (21).

In a large pediatric anesthesia service (approximately 18,000 anesthetics per year), McDonnell et al. sought to improve an overburdened and inefficient M&M system that was associated with a culture of blame and lost educational opportunities (19). M&MCs held following restructuring of the reporting mechanism and focusing case selection on educational potential identified multiple areas for improvement, including situations more commonly associated with adverse events (e.g., fluid management and transfusion, emergent exploratory laparotomies), equipment contributions to error, and wider dissemination of information. These were addressed with targeted educational sessions, equipment changes (e.g., replacement of inaccurate atomizers for local anesthetic delivery with syringes and compatible catheters), and presentation of cases at national meetings and as published case reports, respectively (19).

Similarly, as a result of improved case selection and error analysis, Calder et al. showed that succinct recommendations (“M&M Bottom Lines”) could be generated from M&MCs, providing participants with a memorable message that could improve personal practice and be easily disseminated (e.g., “Find one fracture, look for the next one”) (24).

### Satisfaction and Participation

Mandatory attendance of M&MCs is commonly reported in the literature as a requirement of training (6, 7, 12, 15, 19, 27, 28). In moving to a mandatory M&MC system, McDonnell et al. increased M&MC attendance fivefold (19). Interestingly, this action built the habit of attendance, so that when M&MCs were eventually separated in to their own regular schedule, attendance rates were maintained. Mitchell et al. (15) showed that the adoption of a structured presentation [situation, background, assessment, recommendations (SBAR), presented in detail below] was associated in increased user satisfaction, compared with variable presentation formats decided by individual presenters (17).

### Patient Safety

Several studies have reported improvements in patient safety following the presentation of cases at M&MCs and subsequent changes in patient care and management (12, 13, 22, 25, 26, 29, 30). A common theme of these reports is the clear structure to the M&MC process, though the degree and extent of standardization varied. Some studies focused on identifying and enacting mechanisms for improvement coupled with continuous monitoring of progress (12, 13, 20, 25, 29), while others focused on the care pathway (12, 29), standardized presentation, and error analysis (12, 13).

These varied approaches have resulted in a 50% reduction in malpractice claims (20), improved safety culture and quality of care (20, 25, 29, 30), and reductions in mortality of up to 40% (12, 13, 29). Data should be collected prospectively before as well as after implemented changes in management to properly establish the relationship between enacted changes and outcome. Clinical audit, a core element of clinical governance, is an invaluable tool to monitor adherence to changes in practice and related outcomes (31–34). Furthermore, standardization of data collection is a prerequisite for collaborative efforts to assess the impact of proposed changes in care (35).

Within the broader context of patient safety, M&MCs can be viewed as one of a suite of techniques and tools to report, analyze, and prevent errors (36–39). As such, M&MCs should not be applied in isolation but be included in an organizational approach to error management. It is interesting to note that well-managed M&MCs have the potential to encompass several of the key components of improving patient safety: a reporting system, error analysis using a human factors approach, education, and risk reduction. A discussion of error and patient safety is beyond the scope of this Mini Review. Interested readers are referred to reference texts (36, 38, 40, 41).

## M&MC Format

Publicly disclosing and discussing an adverse event is a difficult process. All M&MCs comprise components that can be optimized to yield the greatest benefit from such a process. The general structure described in this section follows that of two models whose performance has been evaluated prospectively and shown to be effective: the Ottawa M&M model (OM3) and the SBAR model (15, 21, 24). A fictionalized account of a clinical case is used to illustrate the individual components.

### Case Reporting and Selection

Under-reporting of adverse events can stem from lack of awareness of an available reporting system, be it formal or informal, or the inability to submit an anonymous report. Educating new staff members to the existence and use of a reporting system and requiring all submissions be through a single hospital-wide database improves the capture of adverse events, with an increase in the total number of reports and self-reports (19).

In general, all cases of mortality should be reviewed with an M&MC, though this is not always the case (42). In some centers, deaths resulting from the natural progression of a condition are not reviewed (30). In cases where morbidity has occurred as a result of an adverse event, not all cases may progress to an M&MC or meet the threshold for review at an M&MC (7, 19, 24). In large centers, the number of reported cases can outstrip the time and resources available to hold M&MCs. There are several possibilities for handling these cases:

1. Include such cases in the institutional reporting system, where they can serve to highlight a trend of complications or collected by theme (e.g., drug calculation errors) and presented as a group.
2. Hold a smaller M&MC restricted to the discipline in which the error occurred.
3. Address failures in individual performance with the appropriate supervisor. When an error has occurred as a result of a deviation from a well-established procedure and has not resulted in an adverse event, the case may not meet the criteria for presentation at an M&MC. An example is provided below:

A dog was given a 10x intravenous overdose of the alpha-adrenergic agonist dexmedetomidine as a result of a drug calculation error by a veterinary student. Standard practice at the clinic was for all student drug calculations (and injectate volumes) to be checked by a veterinary technician before injection. In this case, the error occurred as standard practice was circumvented with the intention to save time. The error was realized within 3 minutes of the injection occurring, the dog was immediately examined, atipamezole was given and the dog was placed under clinical observation for 6 hours. The case was reviewed the same day with the individuals directly involved and the supervising anesthetist.

Such a near-miss incident (no harm resulted as a result of timely intervention or chance) may not proceed to an M&MC but should be recorded in case a pattern of similar events is occurring and to reinforce individual accountability.

Where case selection is necessary, it should be based on the greatest benefit to future patient safety and educational value (19, 24). Cases should be presented and discussed soon after they occur. There is no evidence in support of a specific time frame, but early presentation reinforces the importance of timely acknowledgment of an adverse event (7, 29). In large centers, the establishment of an M&M committee and coordinators facilitates efficient handling of reports and cases are typically presented within 4–8 weeks of reporting (19, 24, 29).

If there is reluctance to participate in, or convene M&MCs, an option to encourage participation is to initially select cases of near-miss incidents. Selecting such a case is still valuable in terms of improving care and providing education by allowing participants to analyze the factors contributing to the event (see root cause analysis, below), make recommendations to avoid similar incidents and learn from the experience.

### M&MC Duration and Frequency

The duration of M&MCs is often unreported though is likely to be a function of available time (e.g., over lunch) and the number of cases to be discussed. Reported durations have ranged from 20 min (15 min presentation plus 5 min discussion) to over an hour (6, 15, 21, 28, 30, 42, 43). The OM3 and SBAR models last 1 h and 20 min, respectively.

Similarly, frequency of M&MCs is highly variable, with a monthly interval being a common frequency reported in the literature (3, 7, 12, 18–20, 23–25, 28–30, 42, 43).

### Moderator

The moderator should be familiar with the M&MC format, principles of error analysis and have sufficient content expertise to guide the presenter during preparation and the audience in participation and have the authority to establish the desired tone, creating an open, collaborative, and supportive discussion without minimizing or magnifying the error (14, 24, 44).

### Presenter

Typically, M&MCs are presented by a clinician directly involved in the case, though whether this is a trainee or senior clinician may vary depending on the complexity of the case and frequency of presentations (18, 24). In programs where presentations are given by trainees, it is important that senior personnel show support in the form of attendance, setting the appropriate tone for discussion, and sharing their experiences (7). Cases may be presented by someone external to the case though those involved with the case should have the option to present (7, 18). If the responsible clinicians are unable to attend, the tone of the M&MC should be the same as though they were present (7).

### Attendees/Audience

Audience composition is highly variable, though a multidisciplinary approach is strongly favored, as this enriches the discussion and maximizes dissemination of information (7, 18, 28–30, 42, 44). An inclusive approach has been advocated to include care staff, having the added benefit of fostering an open safety culture (12, 29, 30, 45).

### Presentation Format

There are few formats for M&MCs presented in the literature and this has been cited as a limiting factor in maximizing the educational opportunity and unbiased case analysis (7, 15, 18, 21). Consequently, recent work has focused on developing and assessing a standardized M&MC presentation format, the SBAR model (Table 1) (21, 25). Communication using SBAR is an example of situational briefing, to efficiently transfer critical information between team members who may occupy different levels in organization hierarchy (46, 47). As described earlier, the SBAR format has educational benefits for attendees and presenters (15, 21).

**Table 1:** The situation, background, assessment, recommendations (SBAR) presentation format for morbidity and mortality conferences.

SBAR component	Elements
Situation: brief statement of problem	Diagnosis at admission, statement of procedure, and adverse event
Background: clinical information pertinent to adverse event	History, indication for procedure, diagnostic studies, procedural details, timeline of care, description of adverse event (recognition, management, outcome)
Assessment and analysis: evaluation of adverse event (what and why)	What: sequence of events. Why: root cause analysis^a^
Review of the literature: evidence-based practice	Relevant literature
Recommendations: prevention of recurrence	Identify how event could have been prevented or better managed. Identify learning outcomes and recommendations

*Adapted from Mitchell et al. (15, 21)*.

*^a^Numerous methods for root cause analyses exist (see main text)*.

### Root Cause Analysis

**Figure 1:**
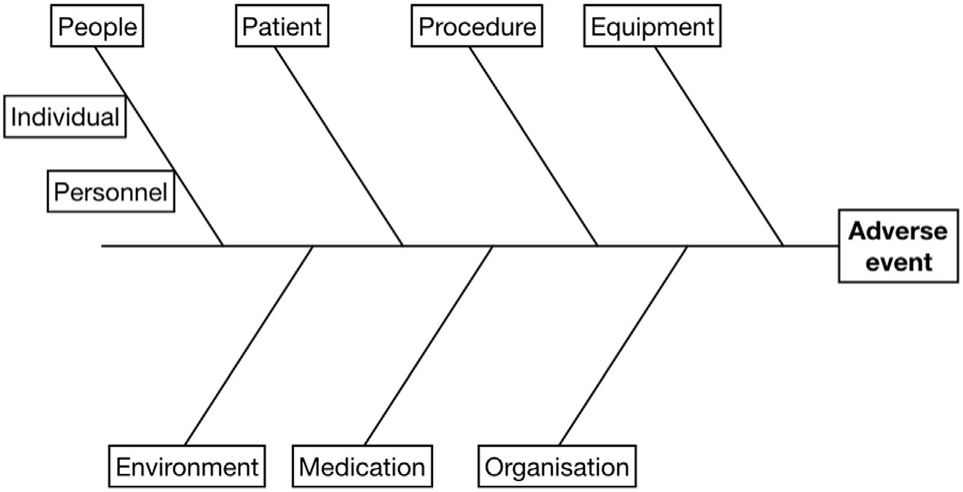
Fishbone diagram used to facilitate root cause analysis. The adverse event is listed at the “head” and potential contributory factors are examined to establish a cause and effect relationship. See Presentations S1 and S2 in Supplementary Material for an example.

The objective of root cause analysis is to identify factors contributing to an adverse event. Several methods have been reported in the M&MC literature, with the common goal of gaining a deeper understanding of the circumstances surrounding an adverse event (13, 15, 21, 25, 43, 45, 48, 49). The method presented here is the fishbone diagram (also known as, cause and effect, Ishikawa, or Fishikawa diagram). It is recommended for process improvement as it provides a visual framework for analysis and discussion and is one of the seven basic quality control tools (Figure 1) (50). The adverse event represents the “head” of the fish and each bone represents a potential contributing factor. The example included in Presentations S1 and S2 in Supplementary Material is based on the work of reason (40, 41) though other, more detailed approaches exist (39). The order and position of individual factors is unrelated to any priority; it may be that a sub-factor is a major contributing factor. A small group discussion may be helpful to determine the role, if any, of each factor, with the group formed by the individual(s) most closely involved with the adverse event and a senior team member with understanding of error analysis. In making these determinations questioning why things occurred, using a “five whys” approach, can be useful (50):

1. Describe the problem.
2. Ask “why” it happened.
3. Continue to ask “why” until the root cause is identified (may take more or less than five “whys”).
4. Maintain a focus on the process and not the personalities.

In maintaining a non-punitive environment for M&MCs, it is critical that causes are based on fact (and evidence) rather than opinion. As illustrated with the sample case (below), there are likely to be multiple factors contributing to an adverse event. Identifying these factors facilitates a complete discussion and identifies potential solutions.

### Follow-up

Where recommendations have resulted in changes in practice, relevant outcomes should be tracked to ensure that changes are beneficial and do not lead to unexpected negative consequences. Tracking has been successfully employed to identify improvements in mortality rates and patient care (as described earlier) (12, 13, 20, 25, 29, 30). Clinical audit is a suitable method for tracking performance that is easy to institute (51). Where a system or infrastructure deficit has been identified as a major contributing factor to the adverse event, the appropriate member of leadership (e.g., hospital director) should be notified so the deficit(s) can be addressed. A recent survey of surgical residency programs registered with the American College of Veterinary Surgeons suggests that in the majority of cases (26/35 programs, survey response rate 32%), discussions do not translate in to implemented changes in practice or changes are not tracked to assess outcome (42).

## Conclusion

Current evidence shows that a structured M&MC with a standardized presentation format and root cause analysis, and tracking of outcomes, serves as a valuable educational experience with the greatest potential to improve patient safety and quality of care. The described approaches can easily be adopted and applied in veterinary medicine.

## Sample Case

### Case Selection

A 10-year old, warmblood mare experienced pronounced acute hypoxemia during recovery from general anesthesia for bilateral thoracic limb magnetic resonance imaging (MRI). This case was selected for presentation at an M&MC as it contained multiple factors contributing to an adverse event and illustrated an important perturbation of normal physiology during anesthesia. Case selection was made following an initial case review between an anesthesia resident and supervisor.

### Presenter and Moderator

The presenter (first year anesthesia resident) was directly involved in the case. The moderator was a senior anesthetist with detailed knowledge of the case.

### Audience

The invited audience included equine interns (mandatory participation), residents (mandatory participation), clinicians, and faculty. All final year veterinary students rotating through the equine hospital and student members of the faculty equine club were also invited. The audience included board-certified internists, theriogenologists, surgeons, and anesthesiologists, and a representative anesthesia technician. Approximately 30 people attended.

### Presentation

The SBAR presentation format was used (Presentations S1 and S2 in Supplementary Material). Educational components were provided by presentation and discussion of the adverse event alongside a brief review of relevant respiratory physiology. Duration was set by the context of inclusion in a weekly graduate trainee seminar series and limited to 15 min presentation followed by 10 min discussion. The proportion of time allocated to the discussion was helpful to explore the contributing factors identified and generate recommendations. To set the tone for the presentation and discussion, the opening and closing presentation slides included a statement of the goal of the M&MC (Presentations S1 and S2 in Supplementary Material).

### Root Cause Analysis (Contributing Factors Corresponding to the Fishbone Diagram Are Italicized)

Specific problem—horse became hypoxemic during recovery (confirmed with arterial blood gas analysis), which potentially began during transfer from MRI.

1. Why? Body position was changed from left to right lateral for transfer to recovery (*procedure*) and ventilation was inadequate (*procedure* or *equipment*) during transfer.
2. Why? There was confusion and unclear communication between different teams (anesthesia, radiology, animal handlers—*people-personnel*) and the endotracheal tube cuff was prematurely deflated limiting efficacy of positive pressure ventilation (*people-individual*).
3. Why? One anesthetist was managing multiple cases on both sides of the hospital (small and large animal, *organization*) and the anesthetist was not present at the start of transfer (*people-individual & personnel*).
4. Why? A second anesthetist was unavailable that morning (*organization*).
5. Why? This was a planned absence with an email circulated to service chiefs notifying them of short-staffing in anesthesia (*organization*).

Identified contributing factors were added to a fishbone diagram in Presentations S1 and S2 in Supplementary Material.

### Follow-up

The equine hospital chief attends all equine M&MCs. Each of the fishbone factors was discussed. The role and responsibilities of different personnel were clarified. Recommendations: (1) A leader is designated to manage transfer. The senior anesthetist is ultimately responsible, but has power to delegate leadership if someone with specific expertise is present, such as senior animal handler. (2) Cases should not be transferred without permission of senior anesthetist. (3) Senior anesthetist has the right to delay or turn away elective cases, and cases may be stopped prematurely in the interests of patient safety. (4) A wider discussion of case transfer, with the potential to introduce a checklist or standard operating protocol, was planned.

## Author Contributions

DP, FR-B, and JP concept and design, drafting and revising, final approval, and accountability for all aspects of the work.

## Conflict of Interest Statement

The authors declare that the research was conducted in the absence of any commercial or financial relationships that could be construed as a potential conflict of interest.

## Supplementary Material

Presentation S1 Example of power point presentation given to accompany sample case (English).

Presentation S2 Example of power point presentation given to accompany sample case (Francais).
