# Supplementary material for: Morbidity and Mortality Conferences: A Mini Review and Illustrated Application in Veterinary Medicine
Source: Front Vet Sci. 2018 Mar 6;5:43. doi: 10.3389/fvets.2018.00043 (PMC5845710; doi:10.3389/fvets.2018.00043)
Supplement: Presentation S1 — Example of power point presentation given to accompany sample case (English). [file presentation_1.pptx]

## Slide 1
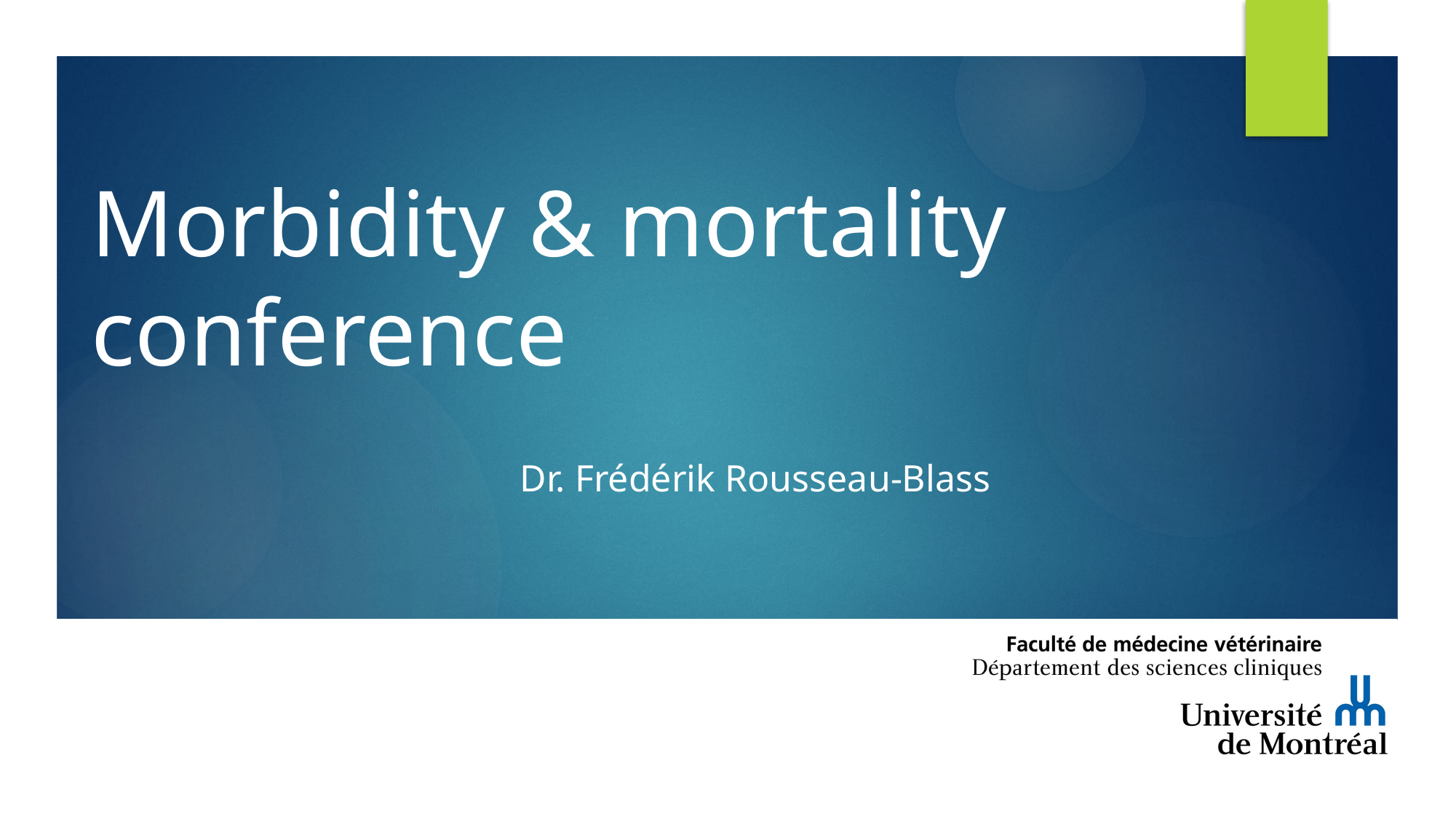

# Morbidity & mortality conference
Dr. Frédérik Rousseau-Blass

## Slide 2
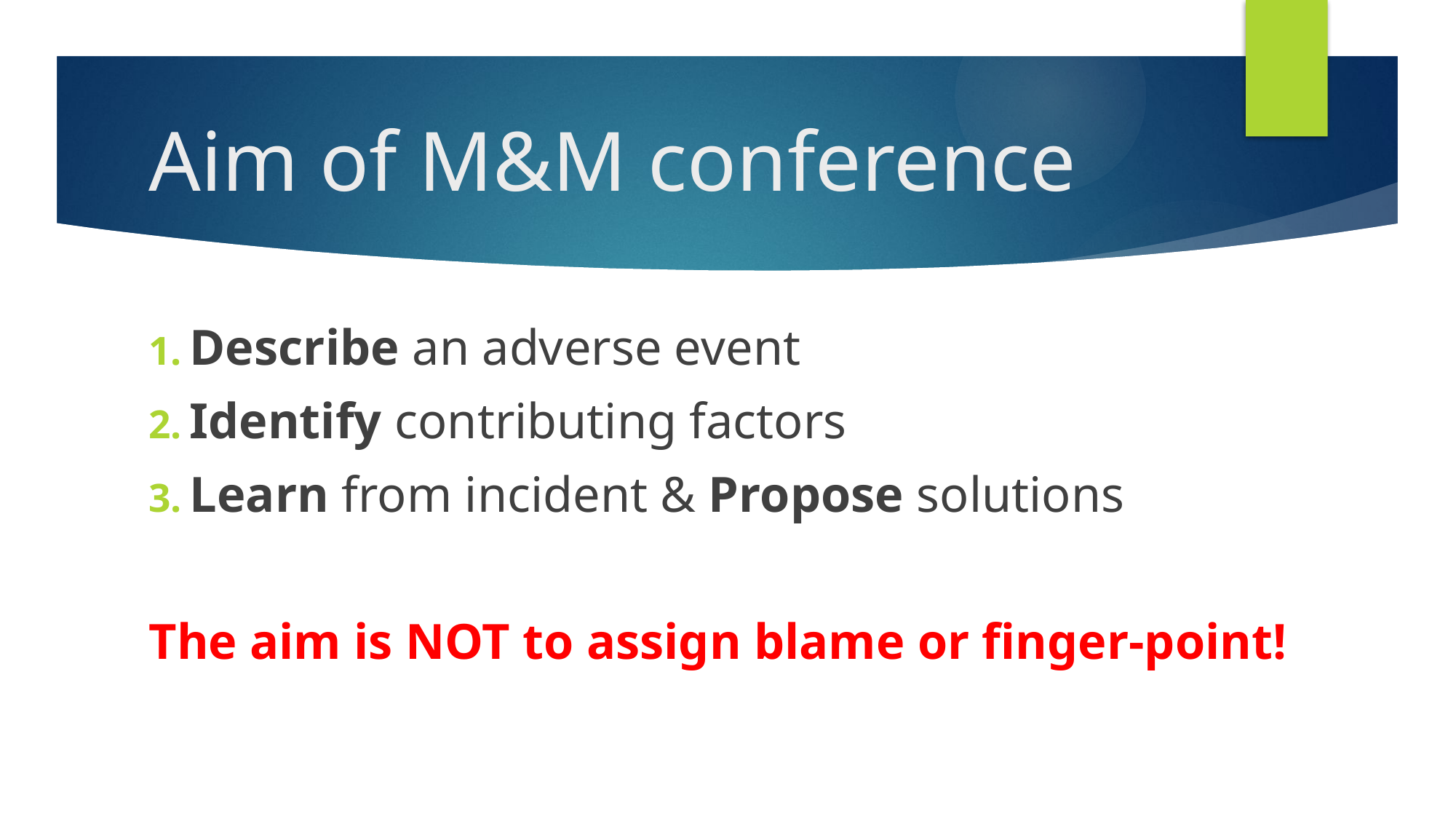

# Aim of M&M conference
Describe an adverse event
Identify contributing factors
Learn from incident & Propose solutions
The aim is NOT to assign blame or finger-point!

## Slide 3
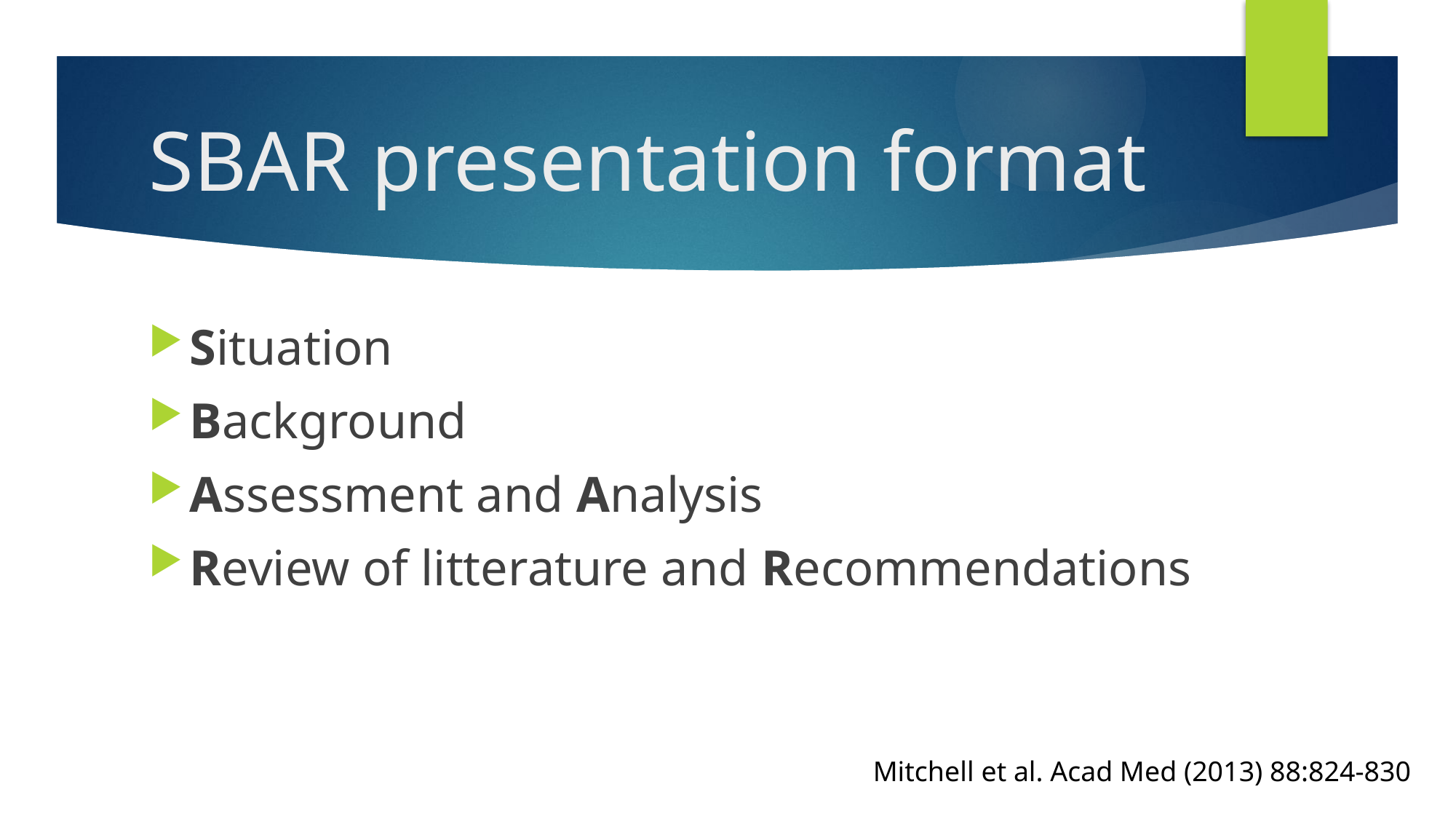

# SBAR presentation format
Situation
Background
Assessment and Analysis
Review of litterature and Recommendations
Mitchell et al. Acad Med (2013) 88:824-830

## Slide 4
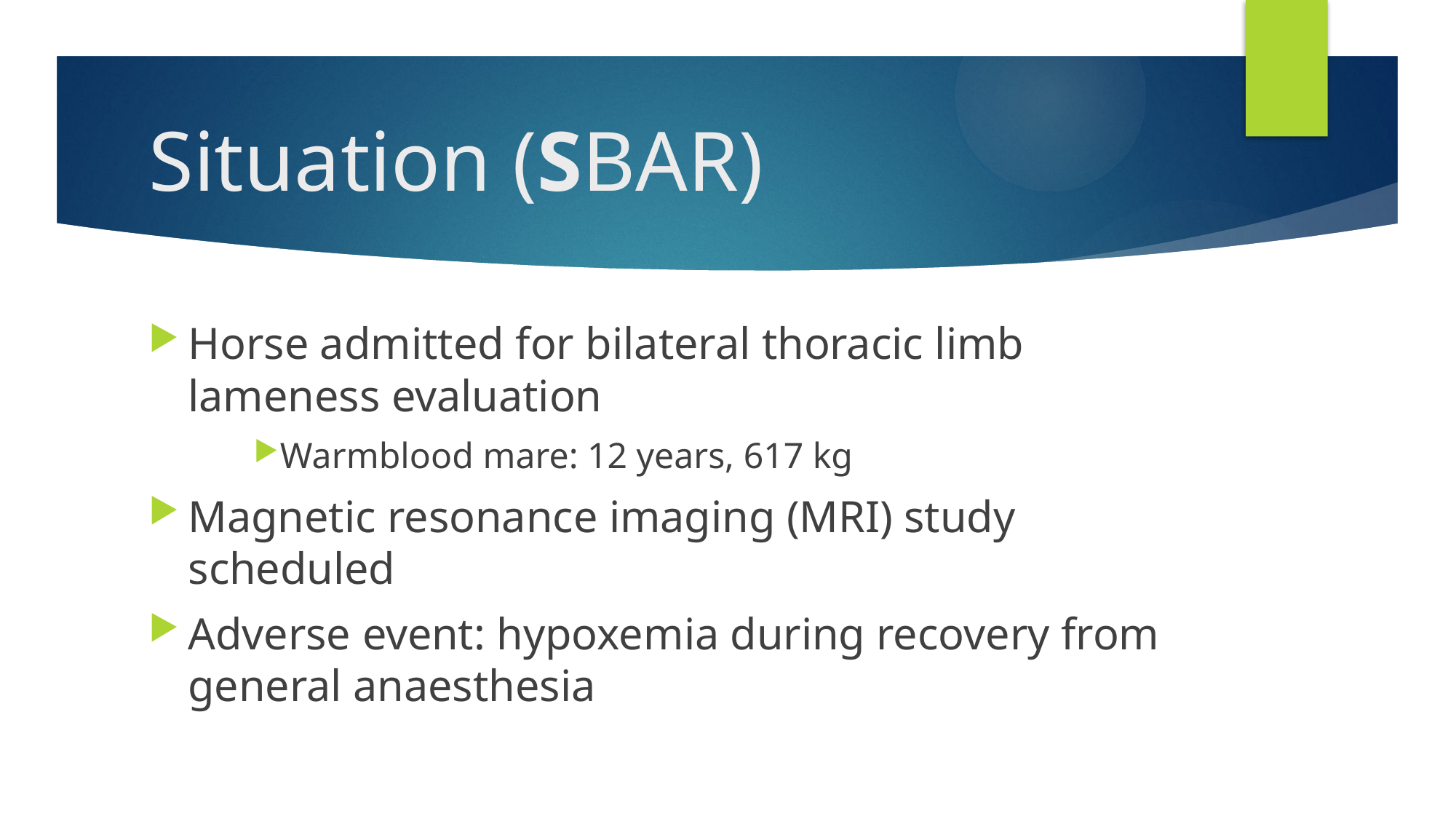

# Situation (SBAR)
Horse admitted for bilateral thoracic limb lameness evaluation
Warmblood mare: 12 years, 617 kg
Magnetic resonance imaging (MRI) study scheduled
Adverse event: hypoxemia during recovery from general anaesthesia

## Slide 5
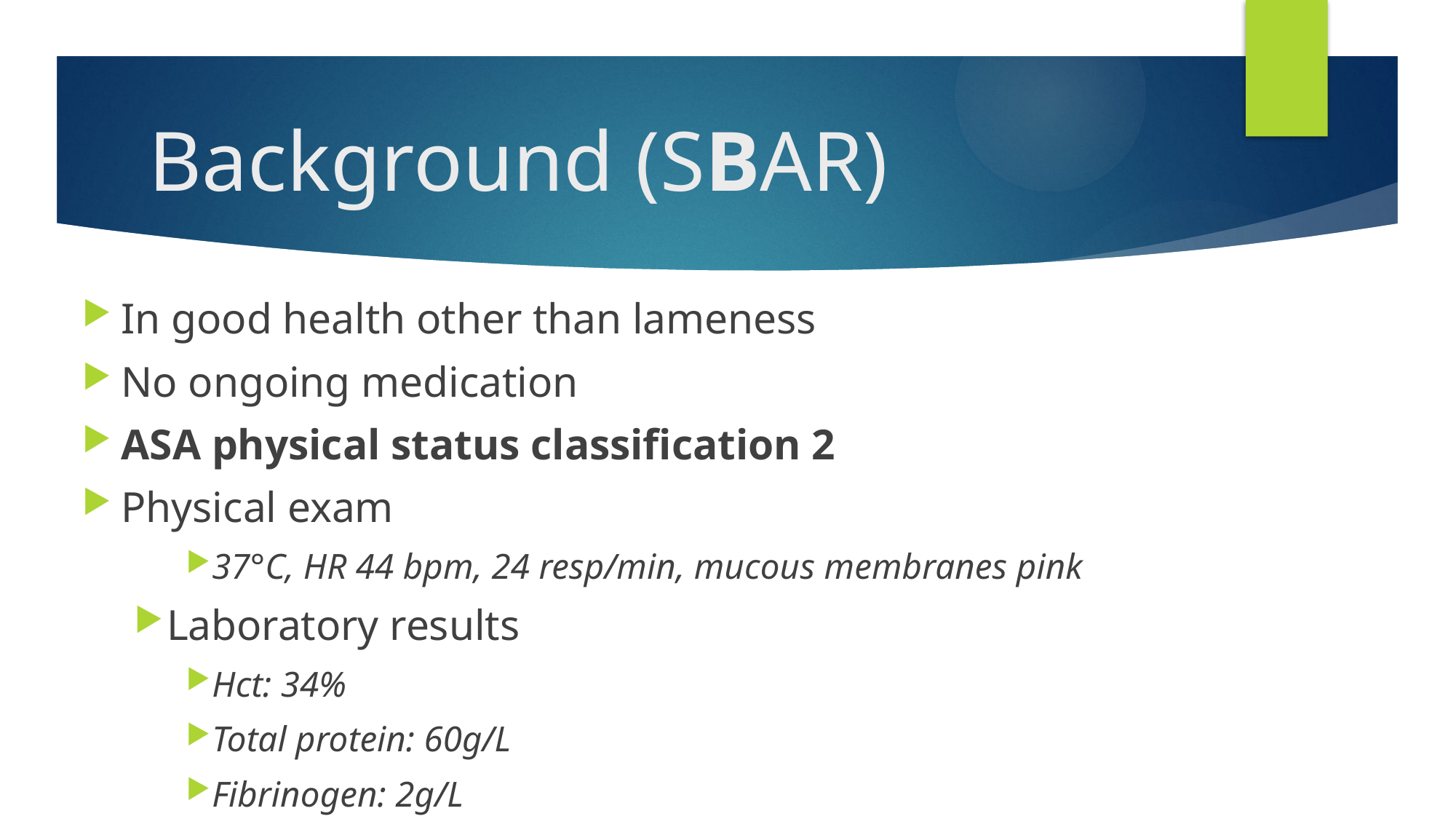

# Background (SBAR)
In good health other than lameness
No ongoing medication
ASA physical status classification 2
Physical exam
37°C, HR 44 bpm, 24 resp/min, mucous membranes pink
Laboratory results
Hct: 34%
Total protein: 60g/L
Fibrinogen: 2g/L

## Slide 6
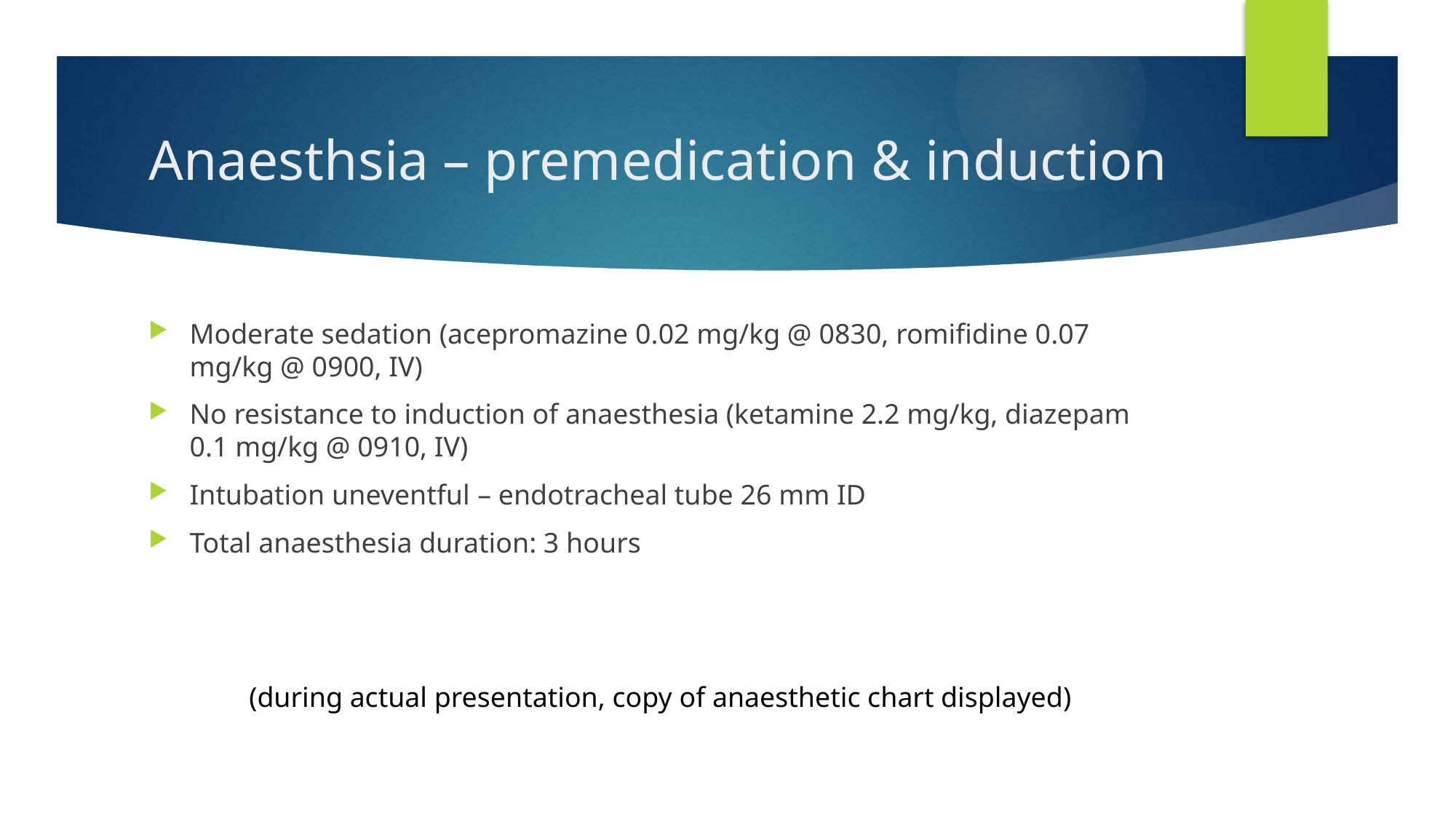

# Anaesthsia – premedication & induction
Moderate sedation (acepromazine 0.02 mg/kg @ 0830, romifidine 0.07 mg/kg @ 0900, IV)
No resistance to induction of anaesthesia (ketamine 2.2 mg/kg, diazepam 0.1 mg/kg @ 0910, IV)
Intubation uneventful – endotracheal tube 26 mm ID
Total anaesthesia duration: 3 hours
(during actual presentation, copy of anaesthetic chart displayed)

## Slide 7
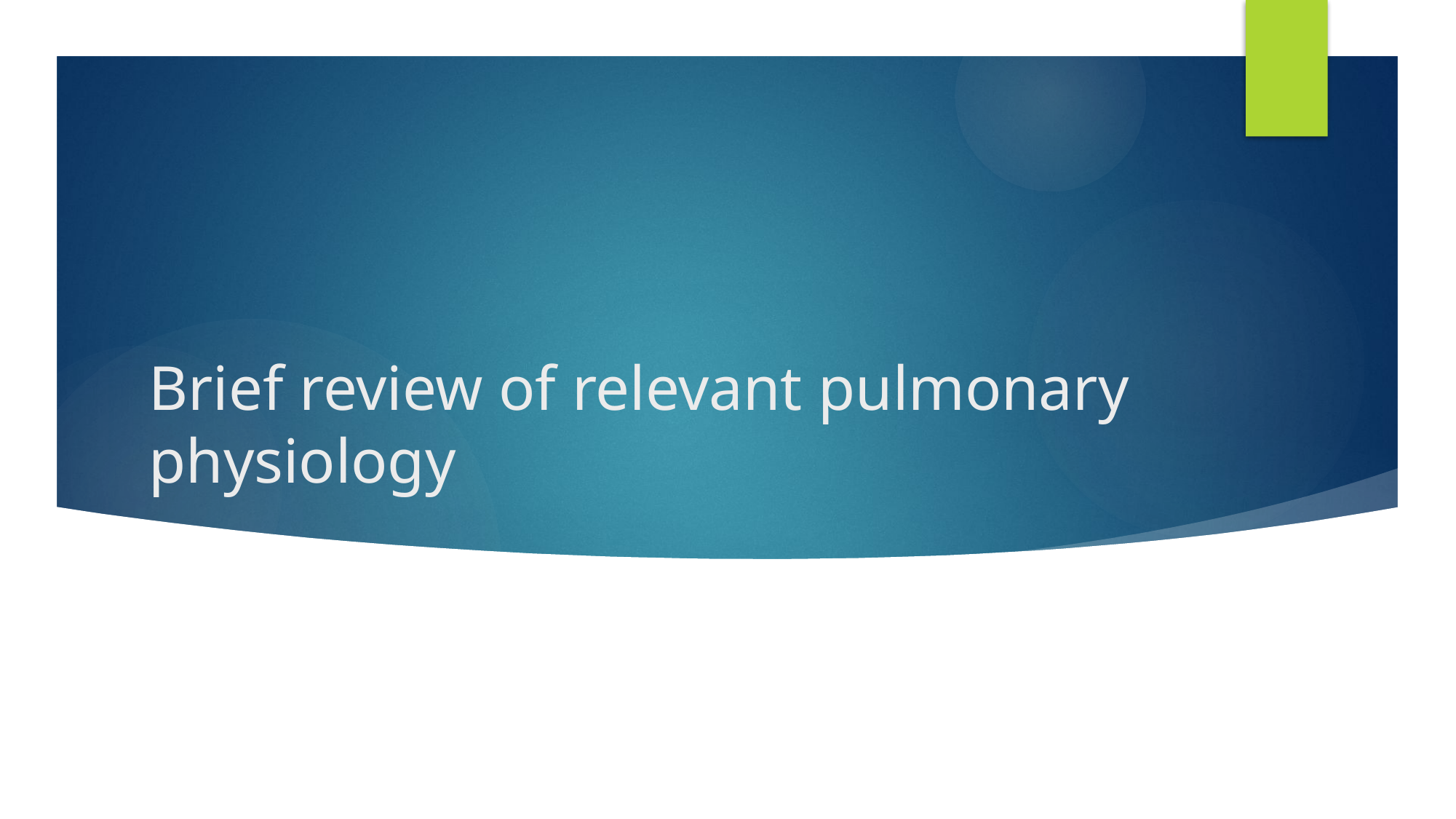

# Brief review of relevant pulmonary physiology

## Slide 8
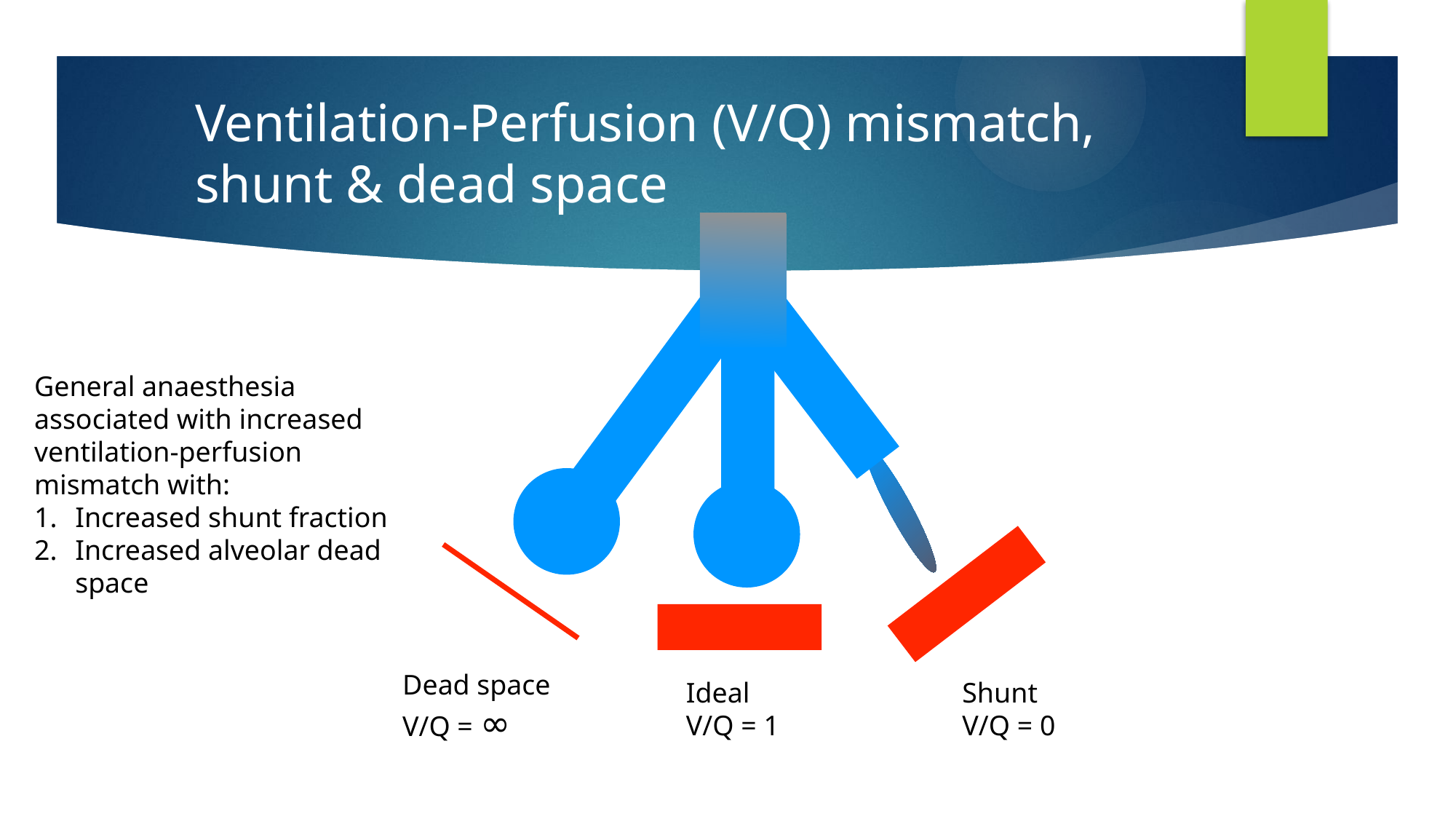

Ventilation-Perfusion (V/Q) mismatch, shunt & dead space
General anaesthesia associated with increased ventilation-perfusion mismatch with:
Increased shunt fraction
Increased alveolar dead space
Dead space
V/Q = ∞
Ideal
V/Q = 1
Shunt
V/Q = 0

## Slide 9
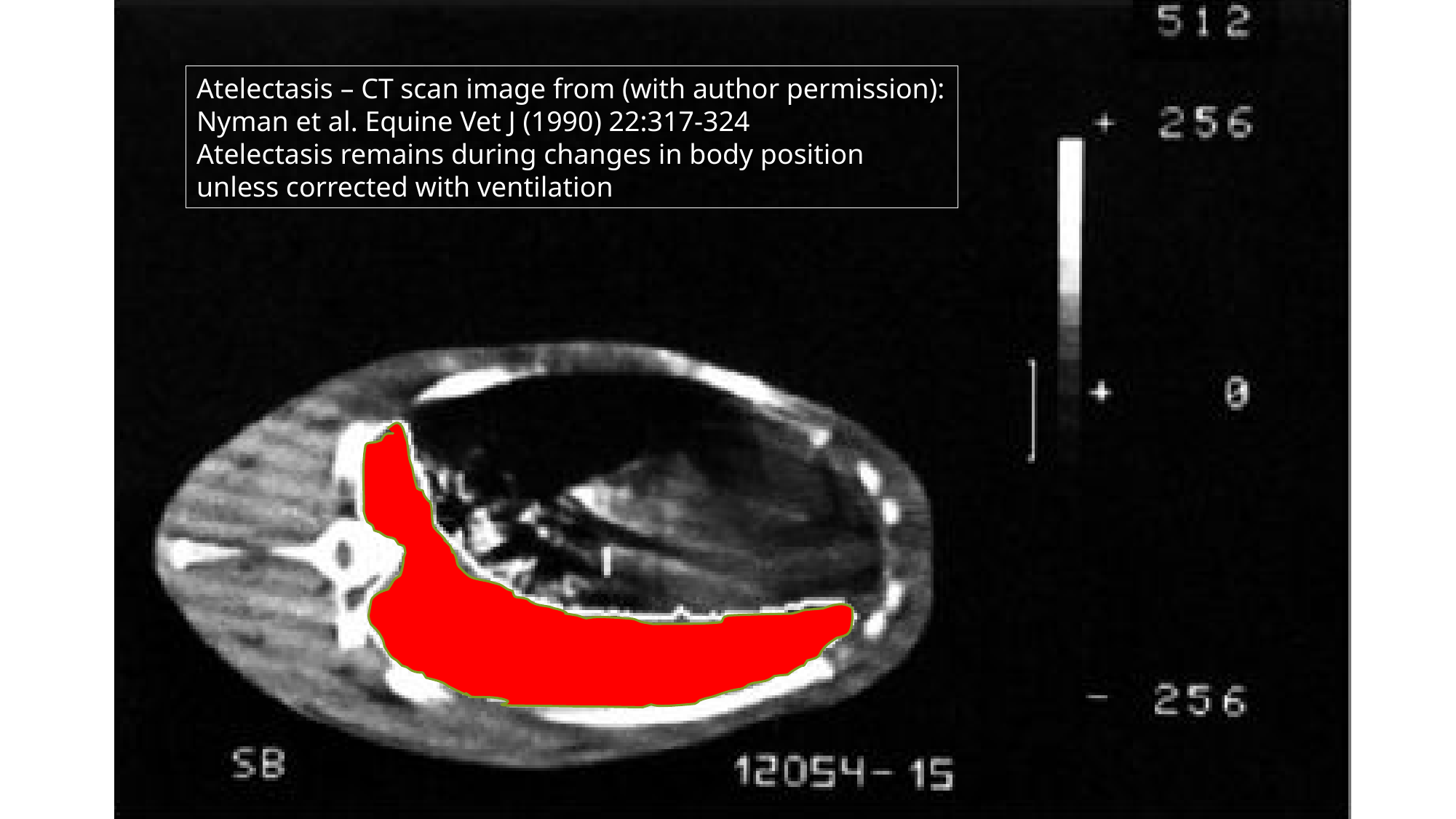

Atelectasis – CT scan image from (with author permission):
Nyman et al. Equine Vet J (1990) 22:317-324
Atelectasis remains during changes in body position unless corrected with ventilation

## Slide 10
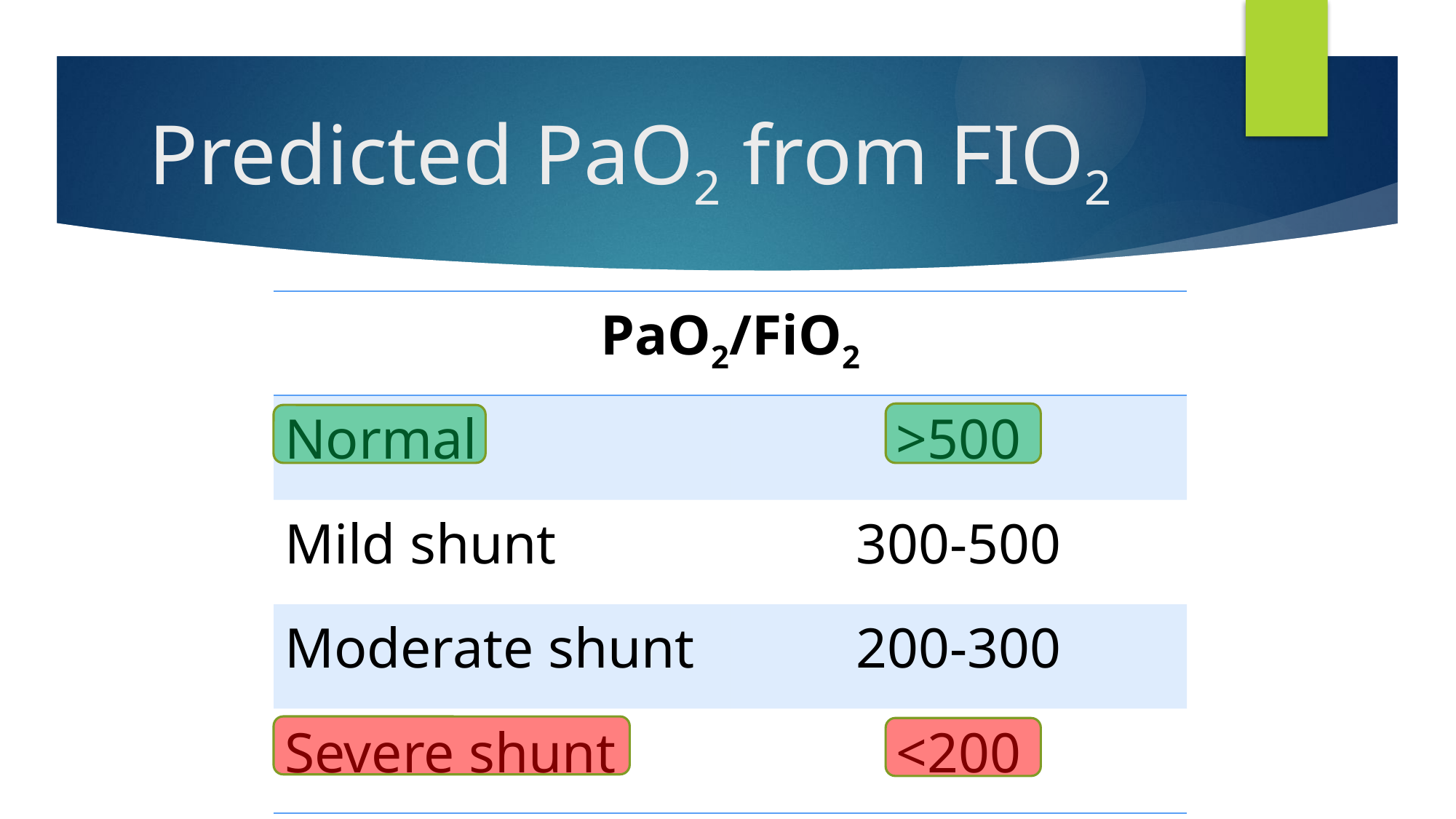

# Predicted PaO2 from FIO2
| PaO2/FiO2 | |
| --- | --- |
| Normal | >500 |
| Mild shunt | 300-500 |
| Moderate shunt | 200-300 |
| Severe shunt | <200 |

## Slide 11
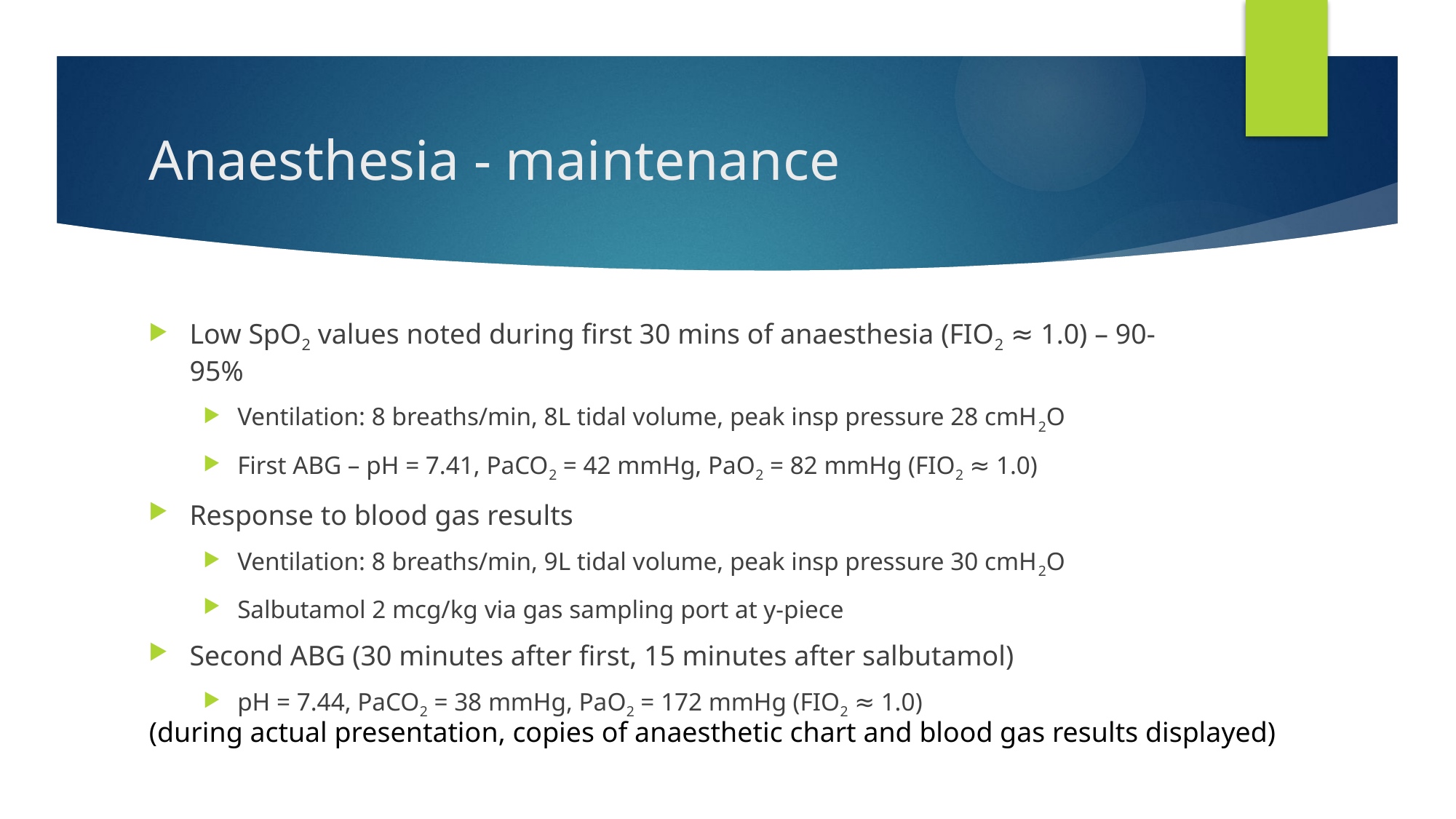

# Anaesthesia - maintenance
Low SpO2 values noted during first 30 mins of anaesthesia (FIO2 ≈ 1.0) – 90-95%
Ventilation: 8 breaths/min, 8L tidal volume, peak insp pressure 28 cmH2O
First ABG – pH = 7.41, PaCO2 = 42 mmHg, PaO2 = 82 mmHg (FIO2 ≈ 1.0)
Response to blood gas results
Ventilation: 8 breaths/min, 9L tidal volume, peak insp pressure 30 cmH2O
Salbutamol 2 mcg/kg via gas sampling port at y-piece
Second ABG (30 minutes after first, 15 minutes after salbutamol)
pH = 7.44, PaCO2 = 38 mmHg, PaO2 = 172 mmHg (FIO2 ≈ 1.0)
(during actual presentation, copies of anaesthetic chart and blood gas results displayed)

## Slide 12
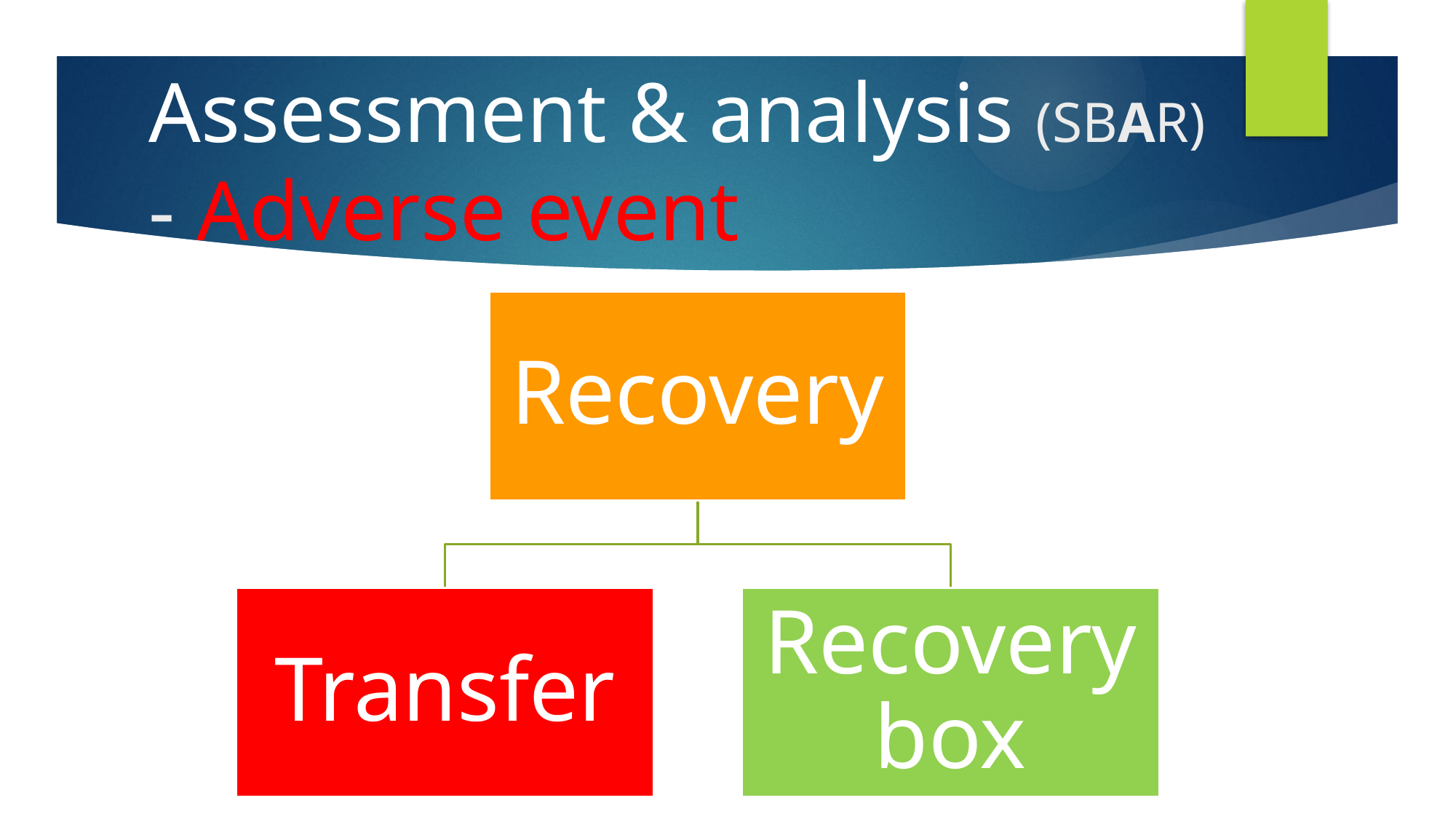

# Assessment & analysis (SBAR) - Adverse event

## Slide 13
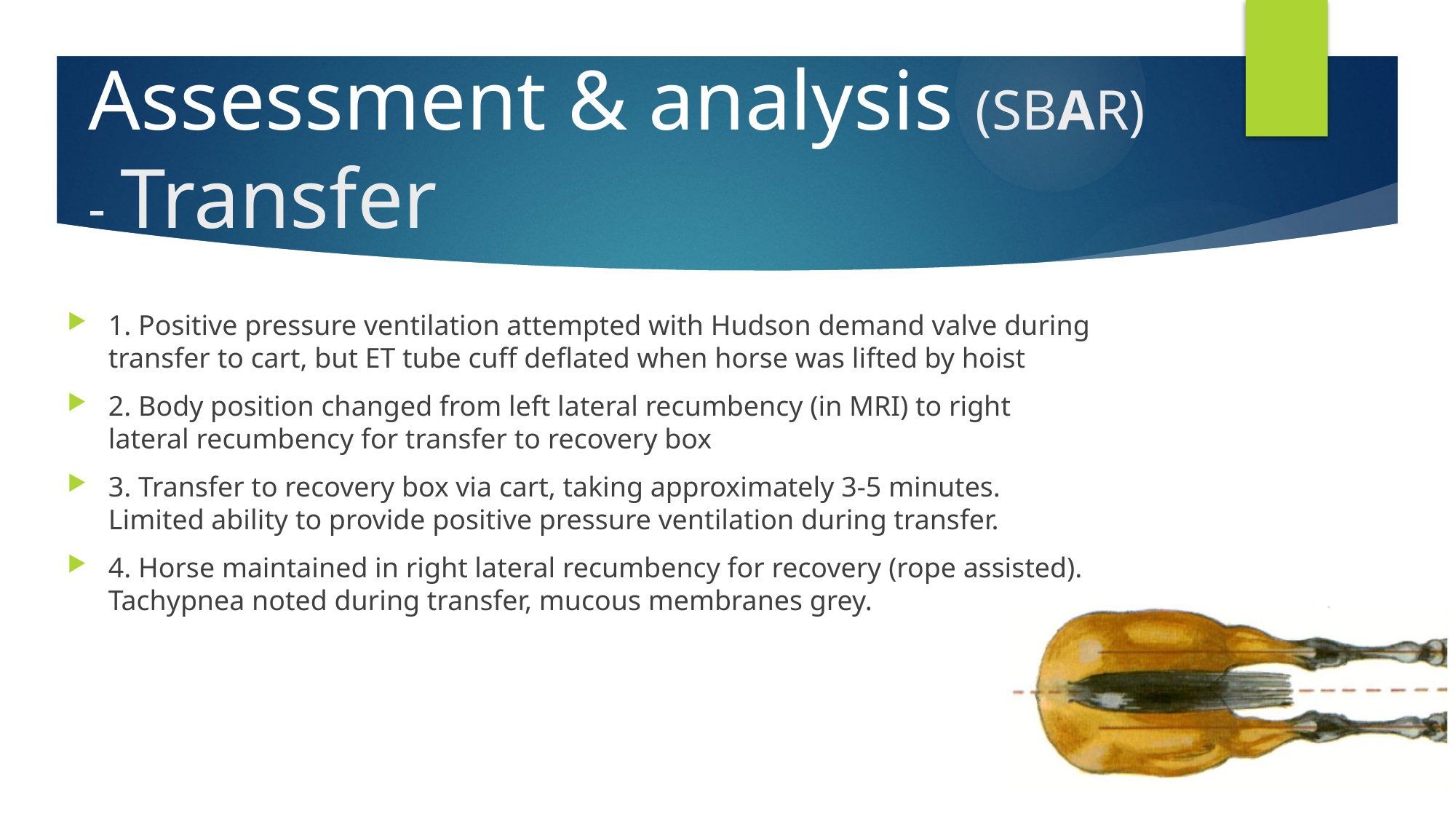

# Assessment & analysis (SBAR) - Transfer
1. Positive pressure ventilation attempted with Hudson demand valve during transfer to cart, but ET tube cuff deflated when horse was lifted by hoist
2. Body position changed from left lateral recumbency (in MRI) to right lateral recumbency for transfer to recovery box
3. Transfer to recovery box via cart, taking approximately 3-5 minutes. Limited ability to provide positive pressure ventilation during transfer.
4. Horse maintained in right lateral recumbency for recovery (rope assisted). Tachypnea noted during transfer, mucous membranes grey.

## Slide 14
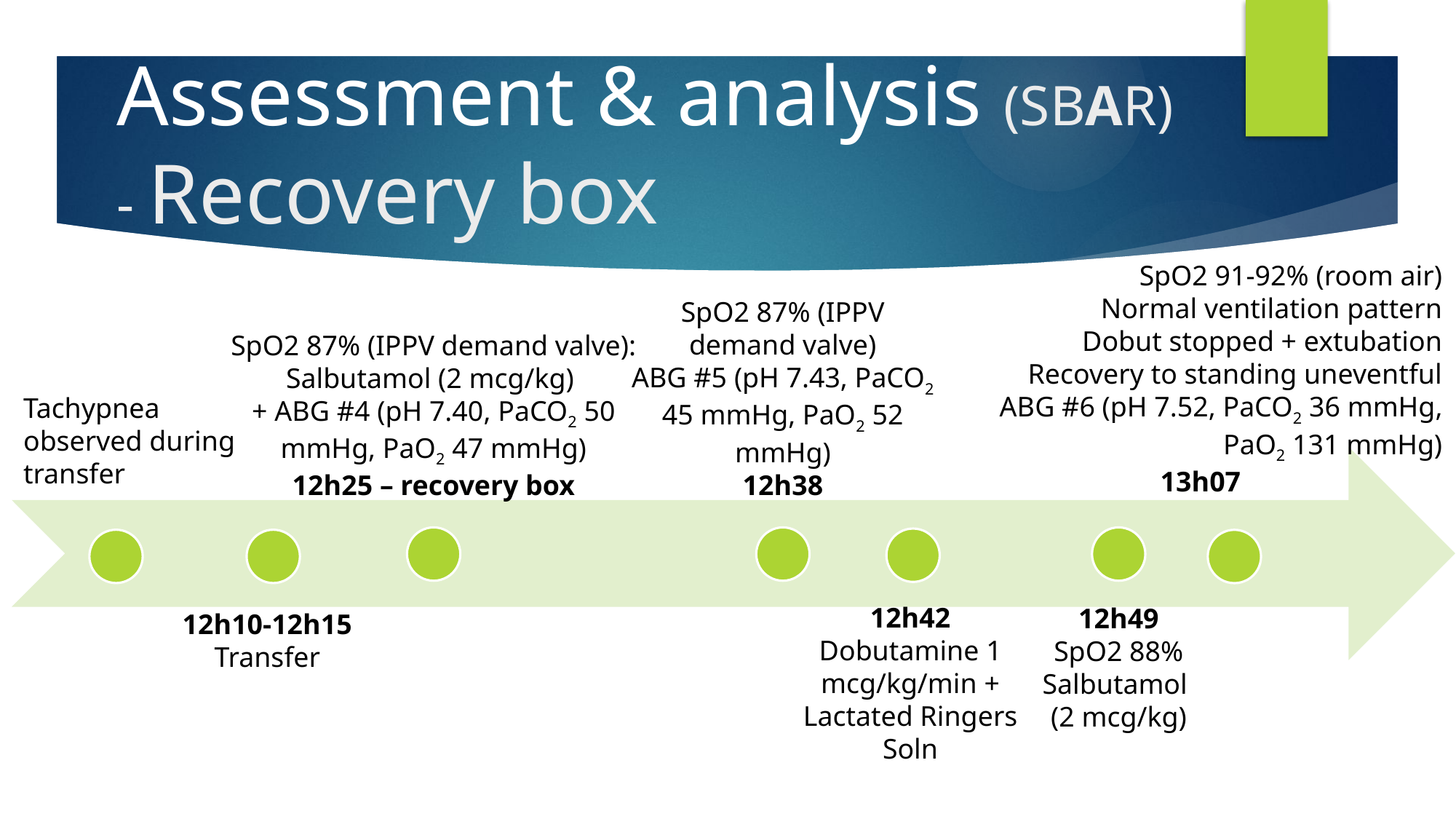

Assessment & analysis (SBAR)
- Recovery box
SpO2 91-92% (room air)
Normal ventilation pattern
Dobut stopped + extubation
Recovery to standing uneventful
ABG #6 (pH 7.52, PaCO2 36 mmHg, PaO2 131 mmHg)
13h07
SpO2 87% (IPPV demand valve)
ABG #5 (pH 7.43, PaCO2 45 mmHg, PaO2 52 mmHg)
12h38
SpO2 87% (IPPV demand valve):
Salbutamol (2 mcg/kg)
+ ABG #4 (pH 7.40, PaCO2 50 mmHg, PaO2 47 mmHg)
12h25 – recovery box
Tachypnea observed during transfer
12h42
Dobutamine 1 mcg/kg/min + Lactated Ringers Soln
12h49
SpO2 88%
Salbutamol
(2 mcg/kg)
12h10-12h15
Transfer

## Slide 15
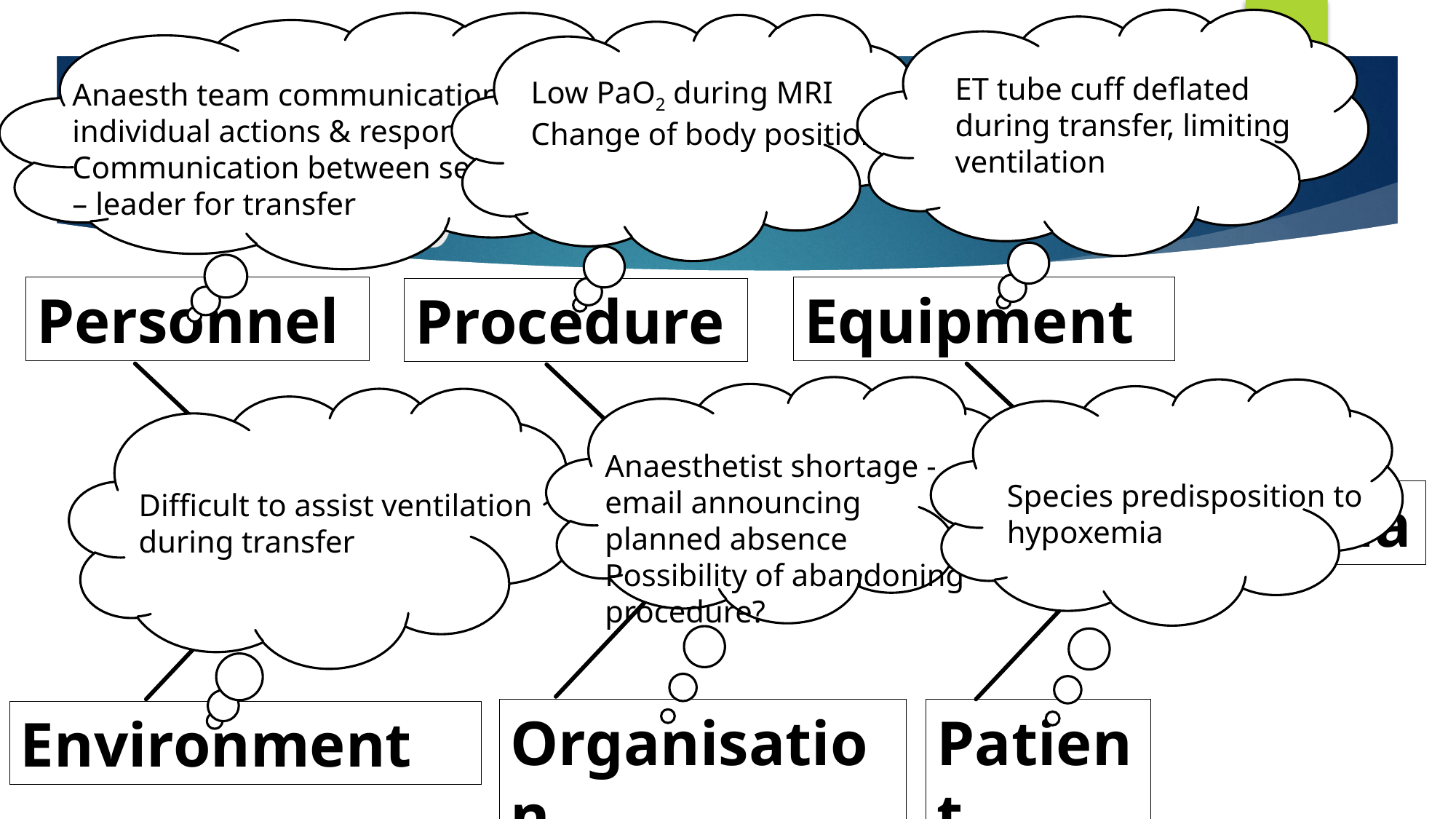

# Assessment & analysis (SBAR) - Recovery box
ET tube cuff deflated during transfer, limiting ventilation
Low PaO2 during MRI
Change of body position
Anaesth team communication – individual actions & responsibilities
Communication between services
– leader for transfer
Personnel
Equipment
Procedure
Anaesthetist shortage - email announcing planned absence
Possibility of abandoning procedure?
Species predisposition to hypoxemia
Difficult to assist ventilation during transfer
Hypoxemia
Patient
Organisation
Environment

## Slide 16
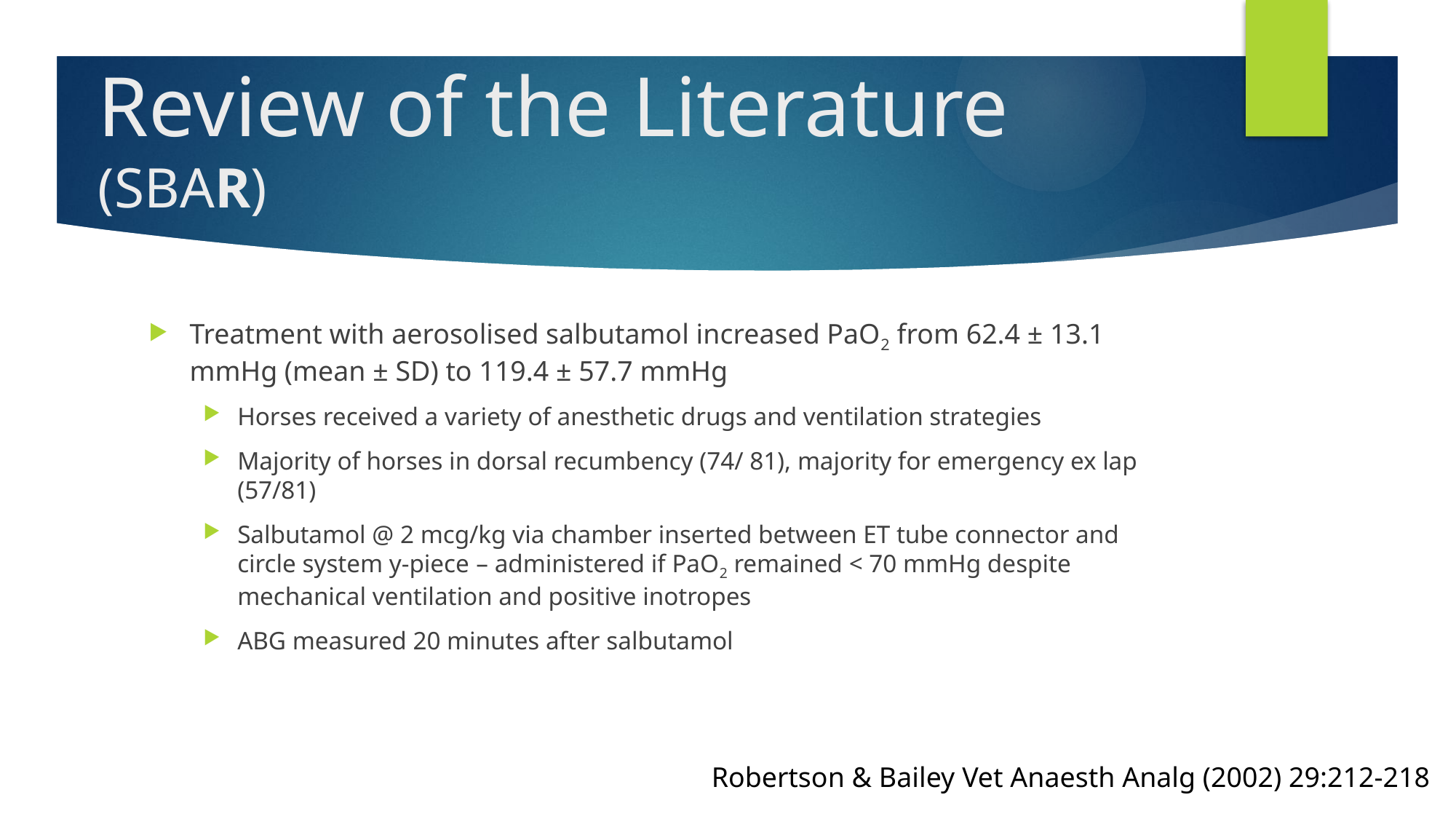

# Review of the Literature (SBAR)
Treatment with aerosolised salbutamol increased PaO2 from 62.4 ± 13.1 mmHg (mean ± SD) to 119.4 ± 57.7 mmHg
Horses received a variety of anesthetic drugs and ventilation strategies
Majority of horses in dorsal recumbency (74/ 81), majority for emergency ex lap (57/81)
Salbutamol @ 2 mcg/kg via chamber inserted between ET tube connector and circle system y-piece – administered if PaO2 remained < 70 mmHg despite mechanical ventilation and positive inotropes
ABG measured 20 minutes after salbutamol
Robertson & Bailey Vet Anaesth Analg (2002) 29:212-218

## Slide 17
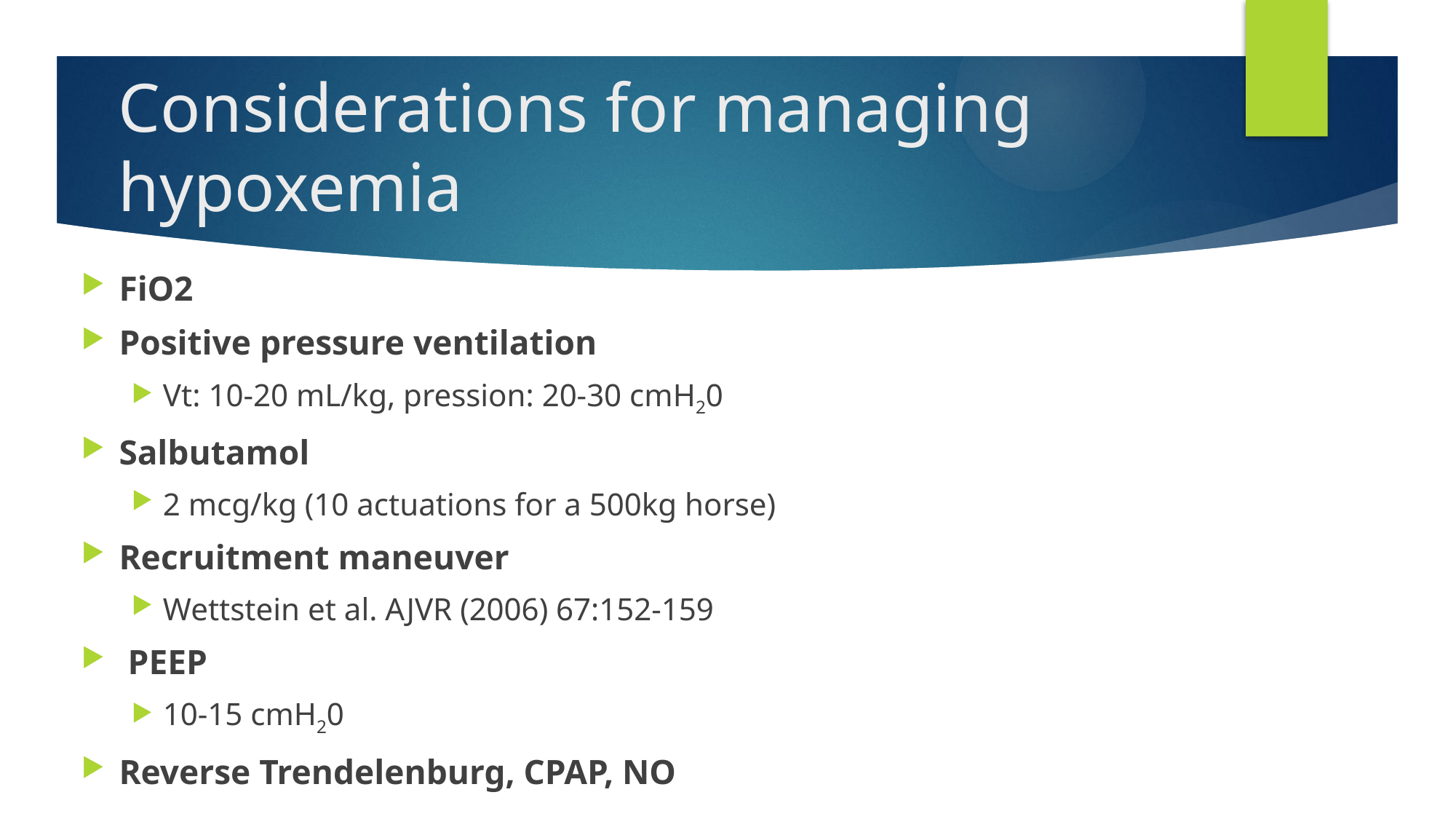

# Considerations for managing hypoxemia
FiO2
Positive pressure ventilation
Vt: 10-20 mL/kg, pression: 20-30 cmH20
Salbutamol
2 mcg/kg (10 actuations for a 500kg horse)
Recruitment maneuver
Wettstein et al. AJVR (2006) 67:152-159
 PEEP
10-15 cmH20
Reverse Trendelenburg, CPAP, NO

## Slide 18
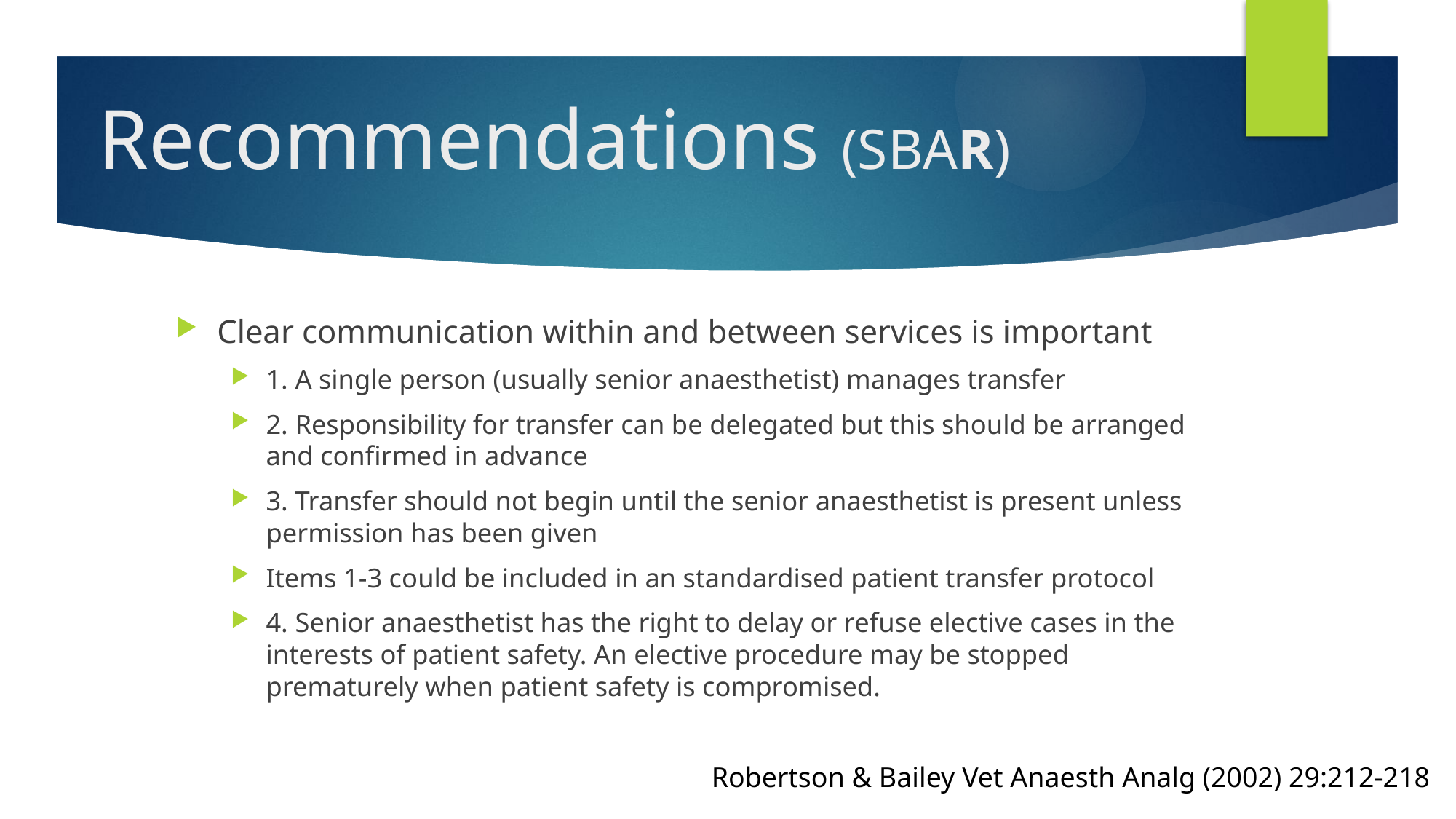

# Recommendations (SBAR)
Clear communication within and between services is important
1. A single person (usually senior anaesthetist) manages transfer
2. Responsibility for transfer can be delegated but this should be arranged and confirmed in advance
3. Transfer should not begin until the senior anaesthetist is present unless permission has been given
Items 1-3 could be included in an standardised patient transfer protocol
4. Senior anaesthetist has the right to delay or refuse elective cases in the interests of patient safety. An elective procedure may be stopped prematurely when patient safety is compromised.
Robertson & Bailey Vet Anaesth Analg (2002) 29:212-218

## Slide 19
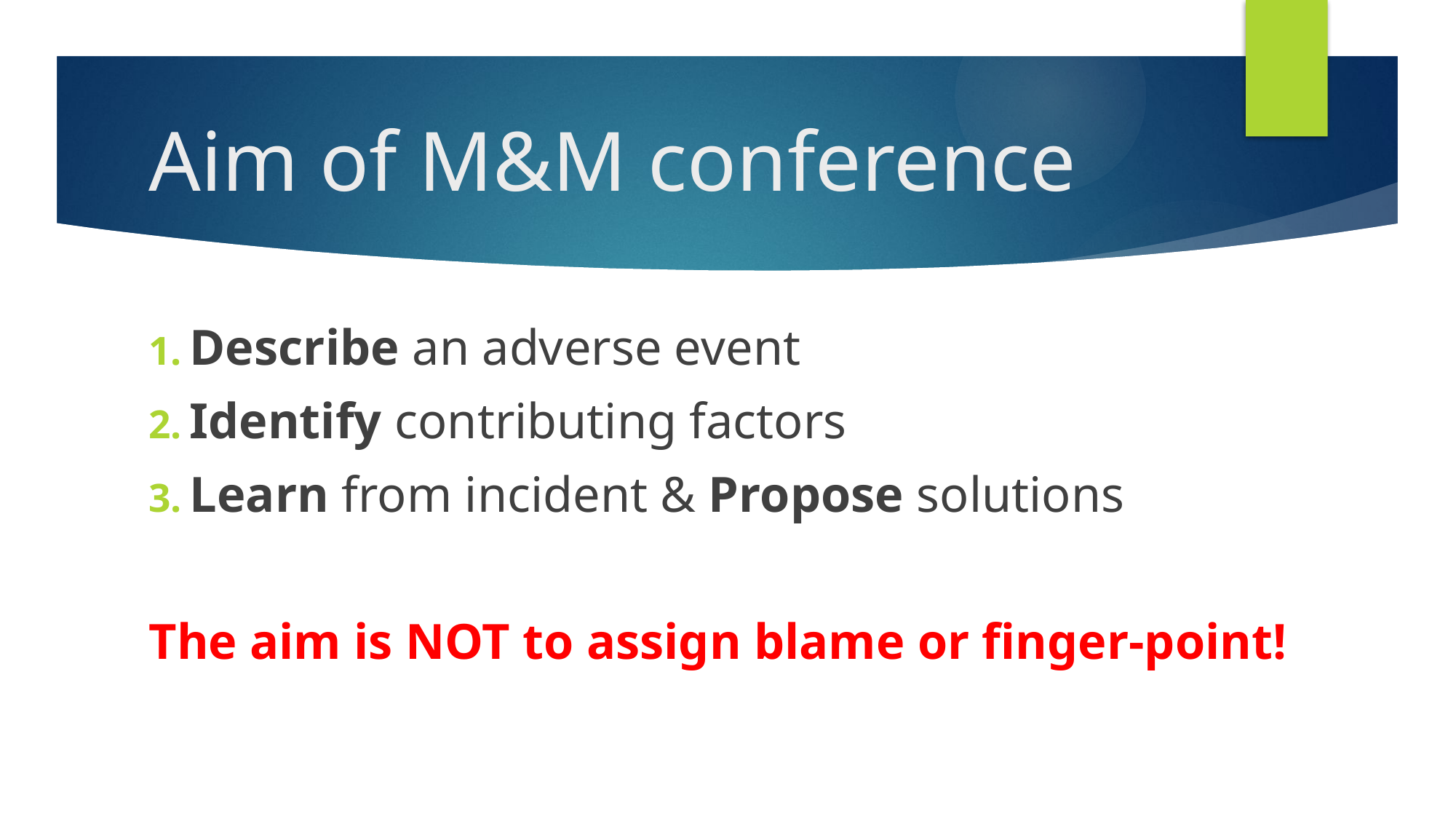

# Aim of M&M conference
Describe an adverse event
Identify contributing factors
Learn from incident & Propose solutions
The aim is NOT to assign blame or finger-point!

## Slide 20
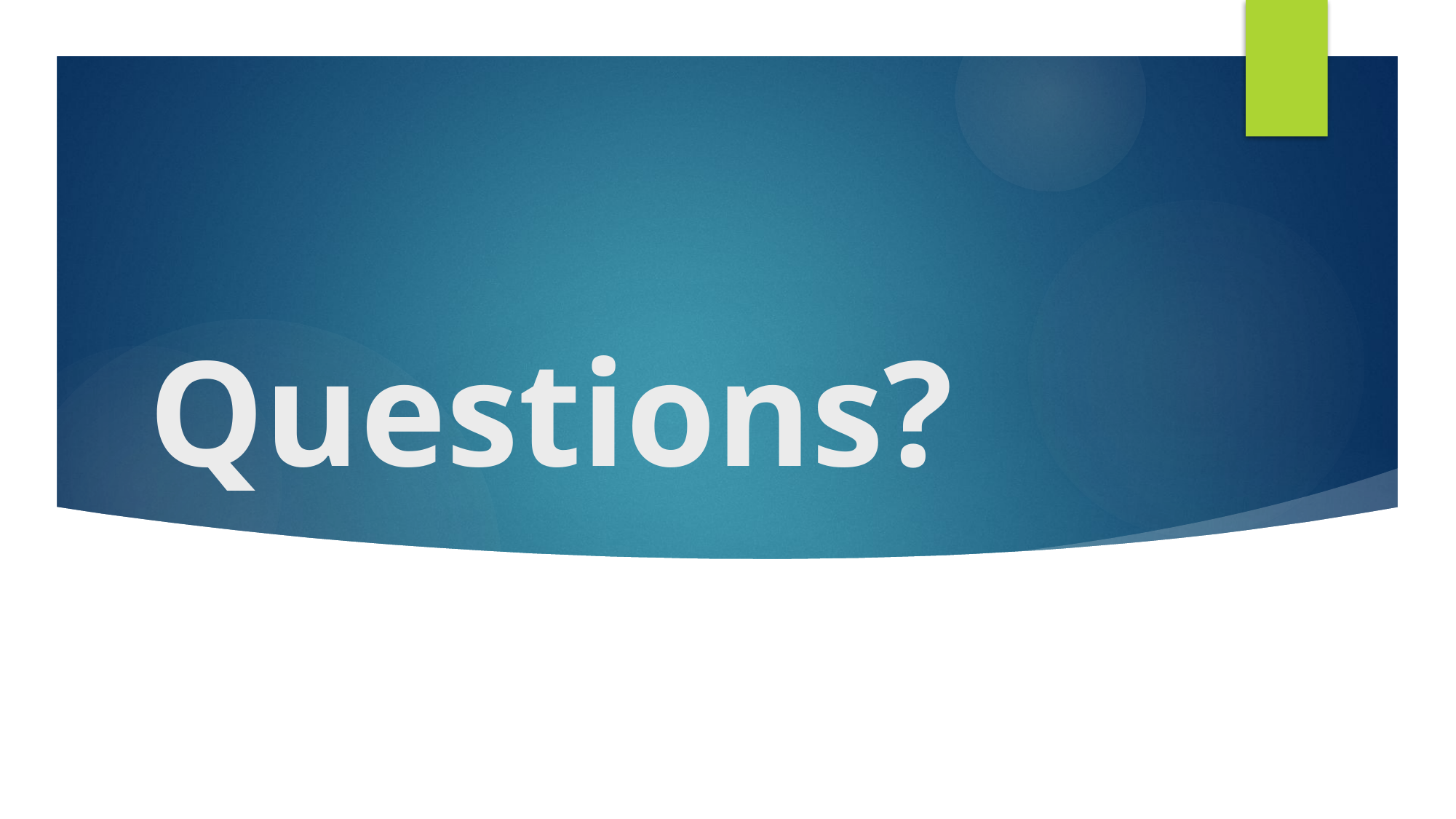

# Questions?
