# Supplementary material for: Morbidity and Mortality Conferences: A Mini Review and Illustrated Application in Veterinary Medicine
Source: Front Vet Sci. 2018 Mar 6;5:43. doi: 10.3389/fvets.2018.00043 (PMC5845710; doi:10.3389/fvets.2018.00043)
Supplement: Presentation S2 — Example of power point presentation given to accompany sample case (Francais). [file presentation_2.pptx]

## Slide 1
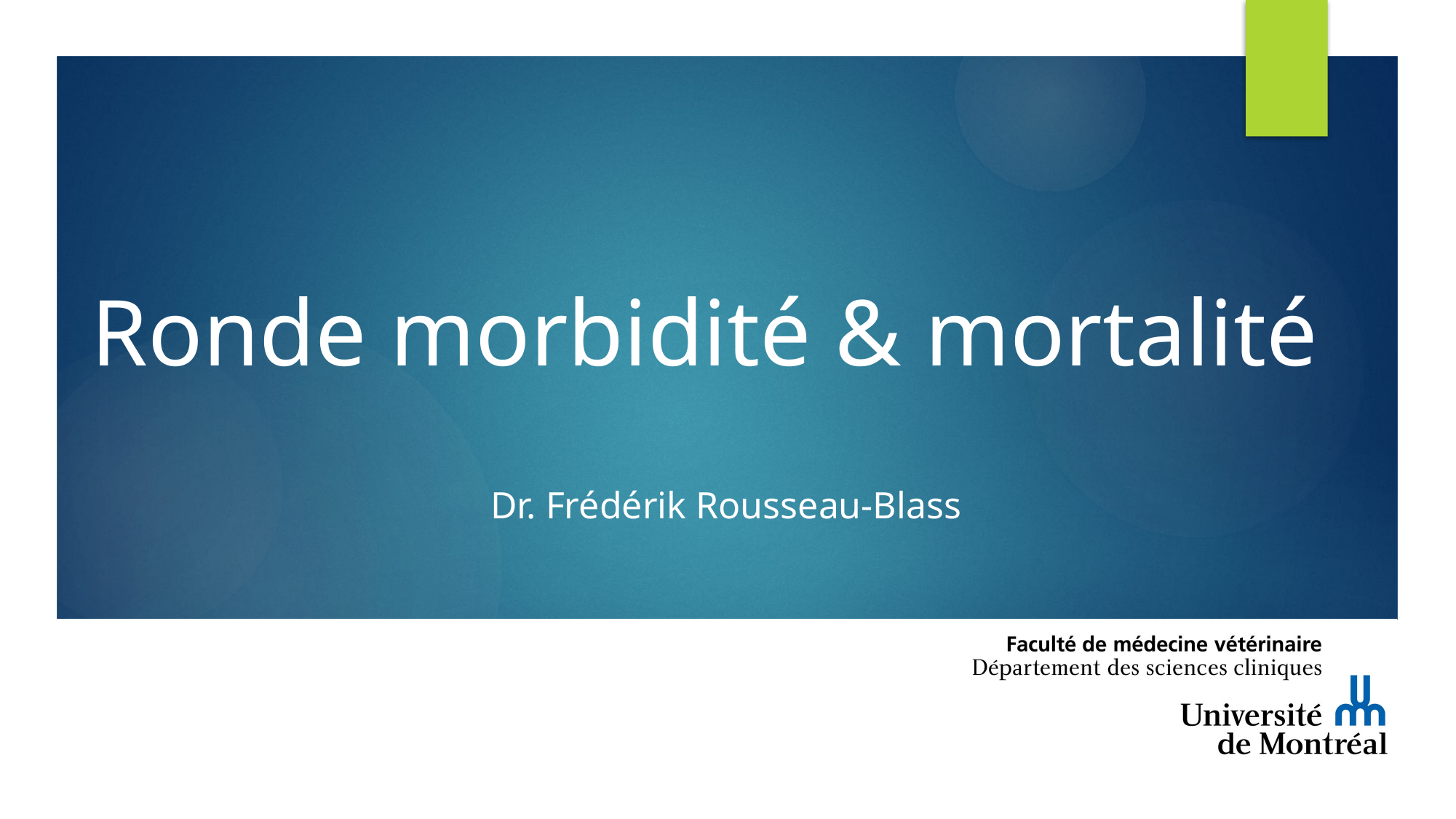

# Ronde morbidité & mortalité
Dr. Frédérik Rousseau-Blass

## Slide 2
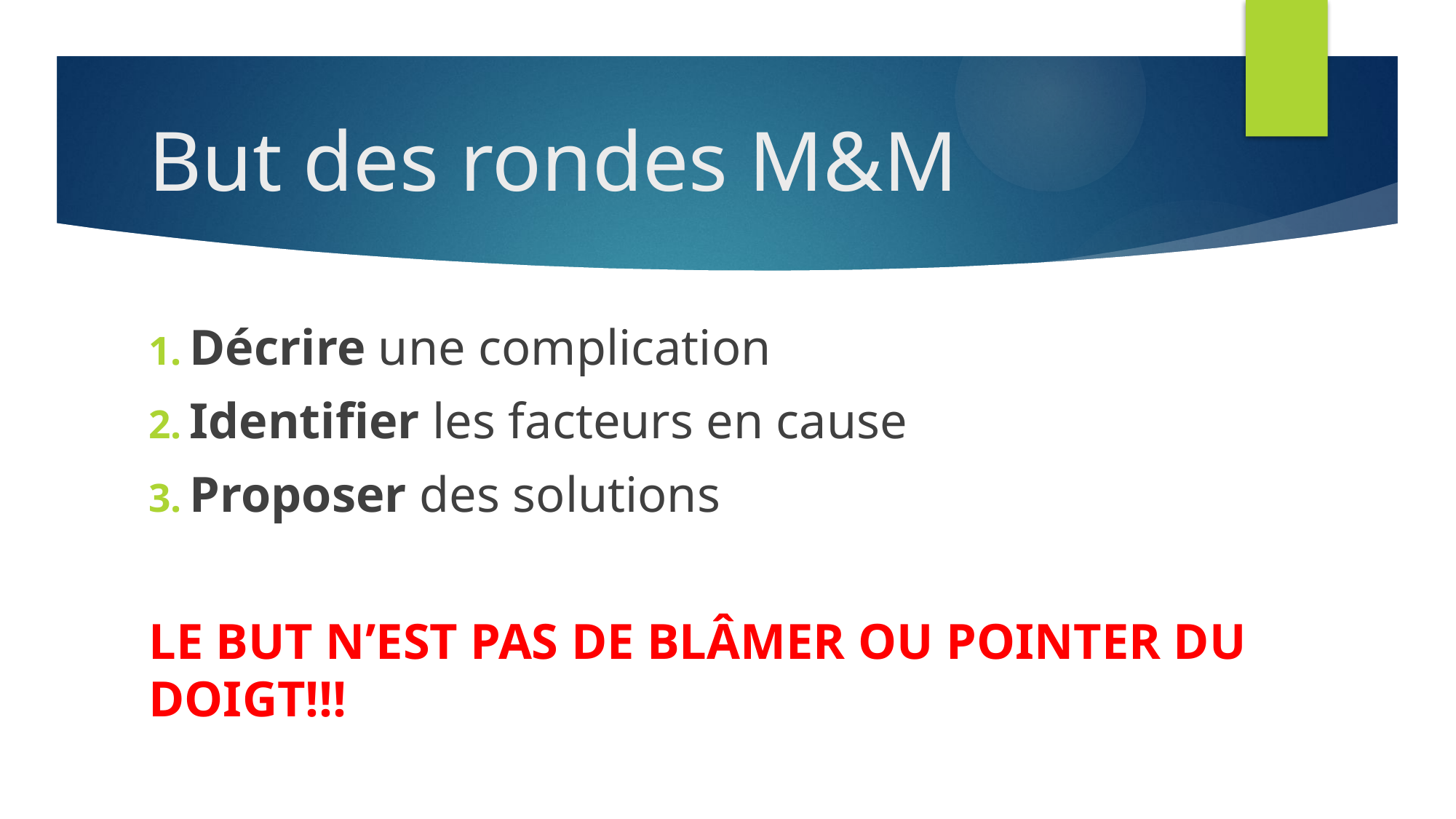

# But des rondes M&M
Décrire une complication
Identifier les facteurs en cause
Proposer des solutions
LE BUT N’EST PAS DE BLÂMER OU POINTER DU DOIGT!!!

## Slide 3
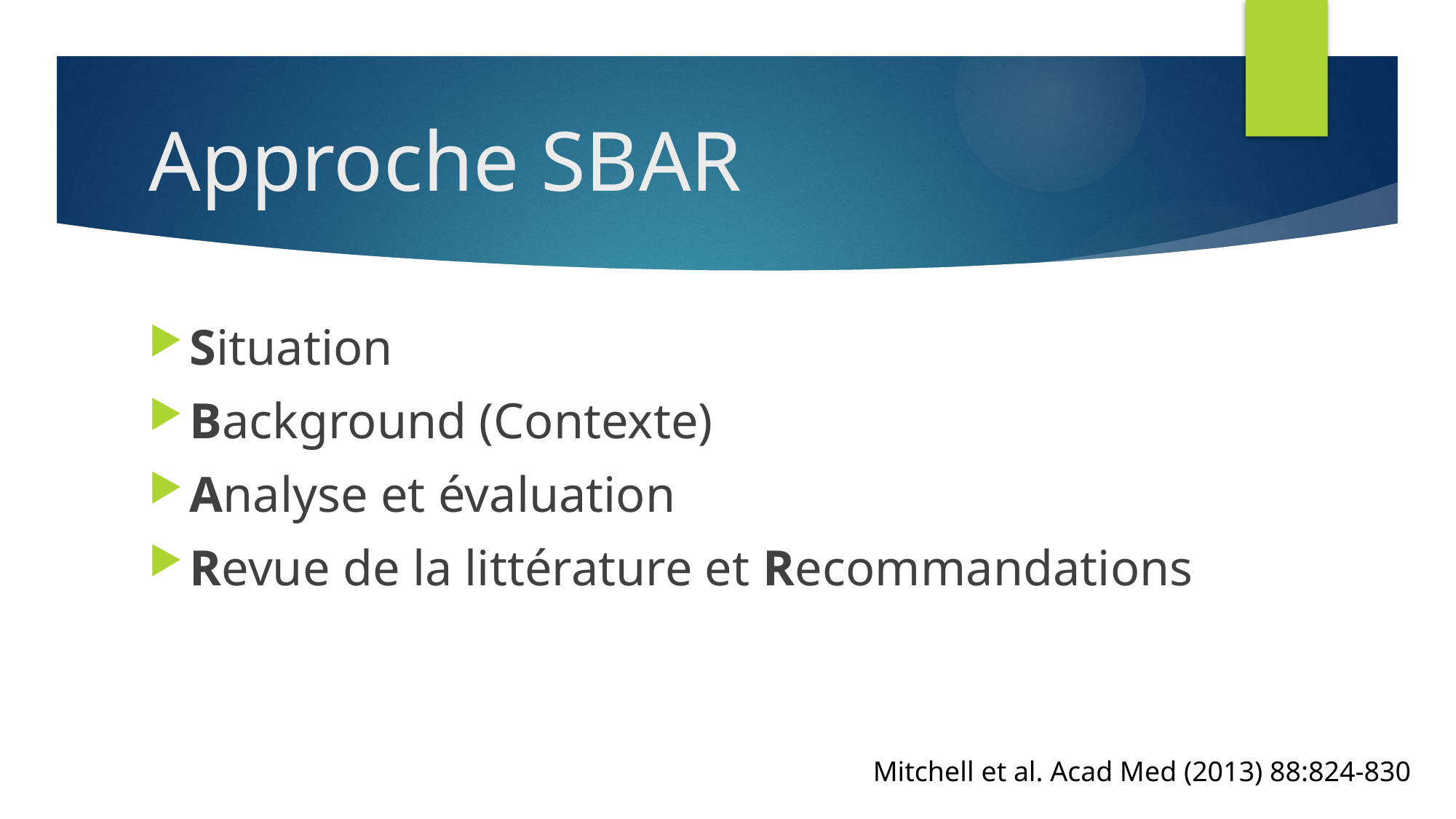

# Approche SBAR
Situation
Background (Contexte)
Analyse et évaluation
Revue de la littérature et Recommandations
Mitchell et al. Acad Med (2013) 88:824-830

## Slide 4
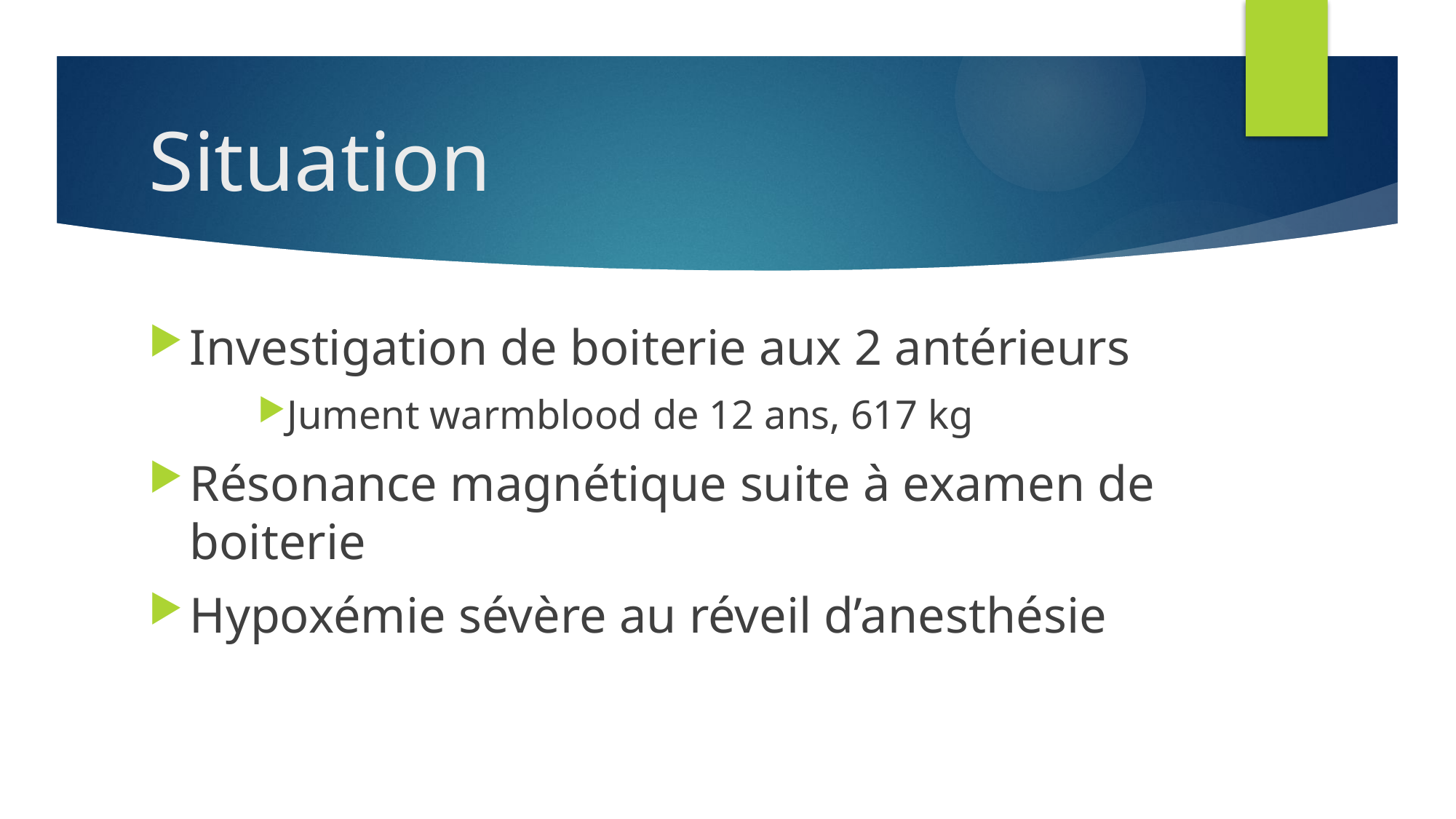

# Situation
Investigation de boiterie aux 2 antérieurs
Jument warmblood de 12 ans, 617 kg
Résonance magnétique suite à examen de boiterie
Hypoxémie sévère au réveil d’anesthésie

## Slide 5
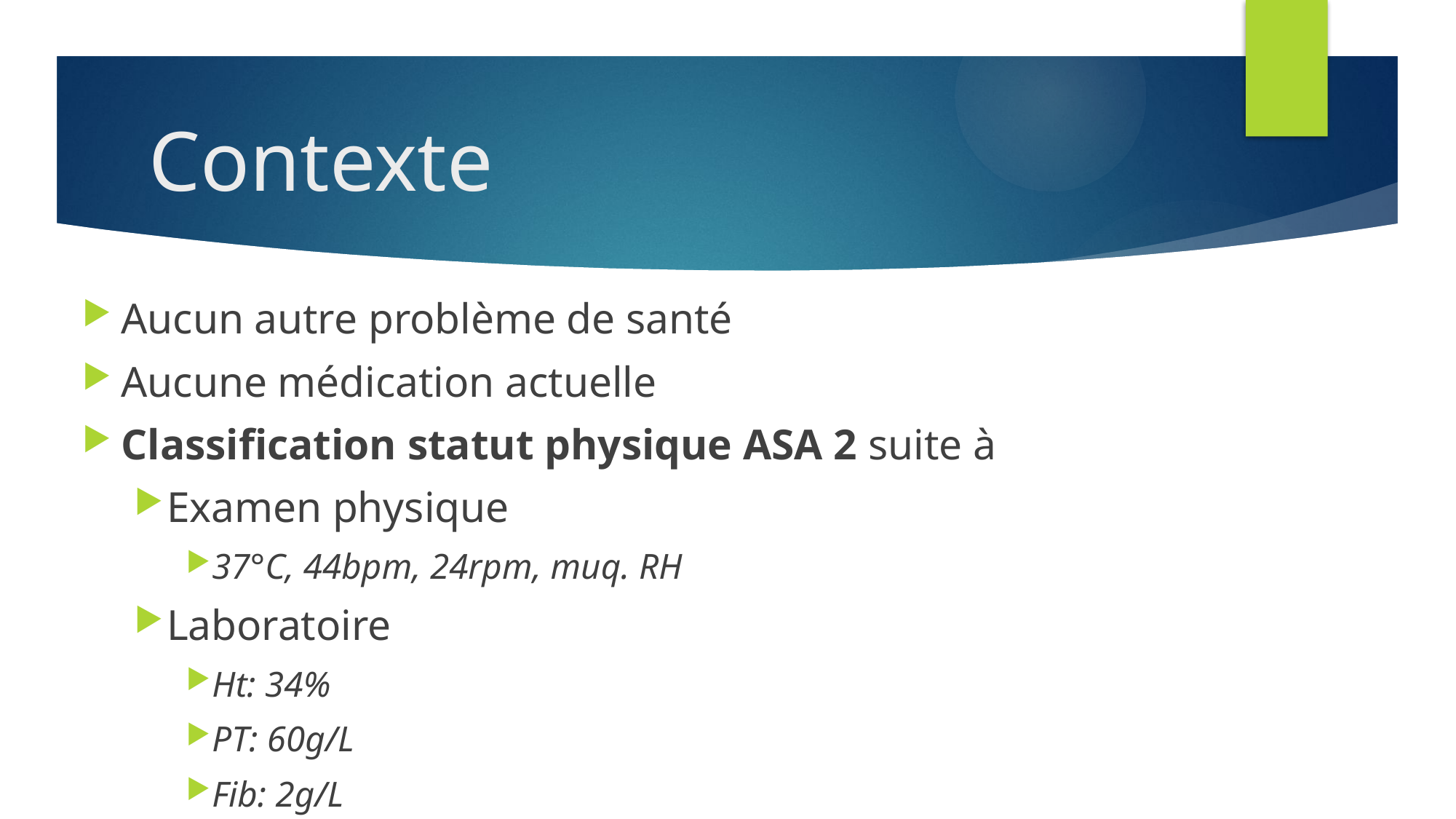

# Contexte
Aucun autre problème de santé
Aucune médication actuelle
Classification statut physique ASA 2 suite à
Examen physique
37°C, 44bpm, 24rpm, muq. RH
Laboratoire
Ht: 34%
PT: 60g/L
Fib: 2g/L

## Slide 6
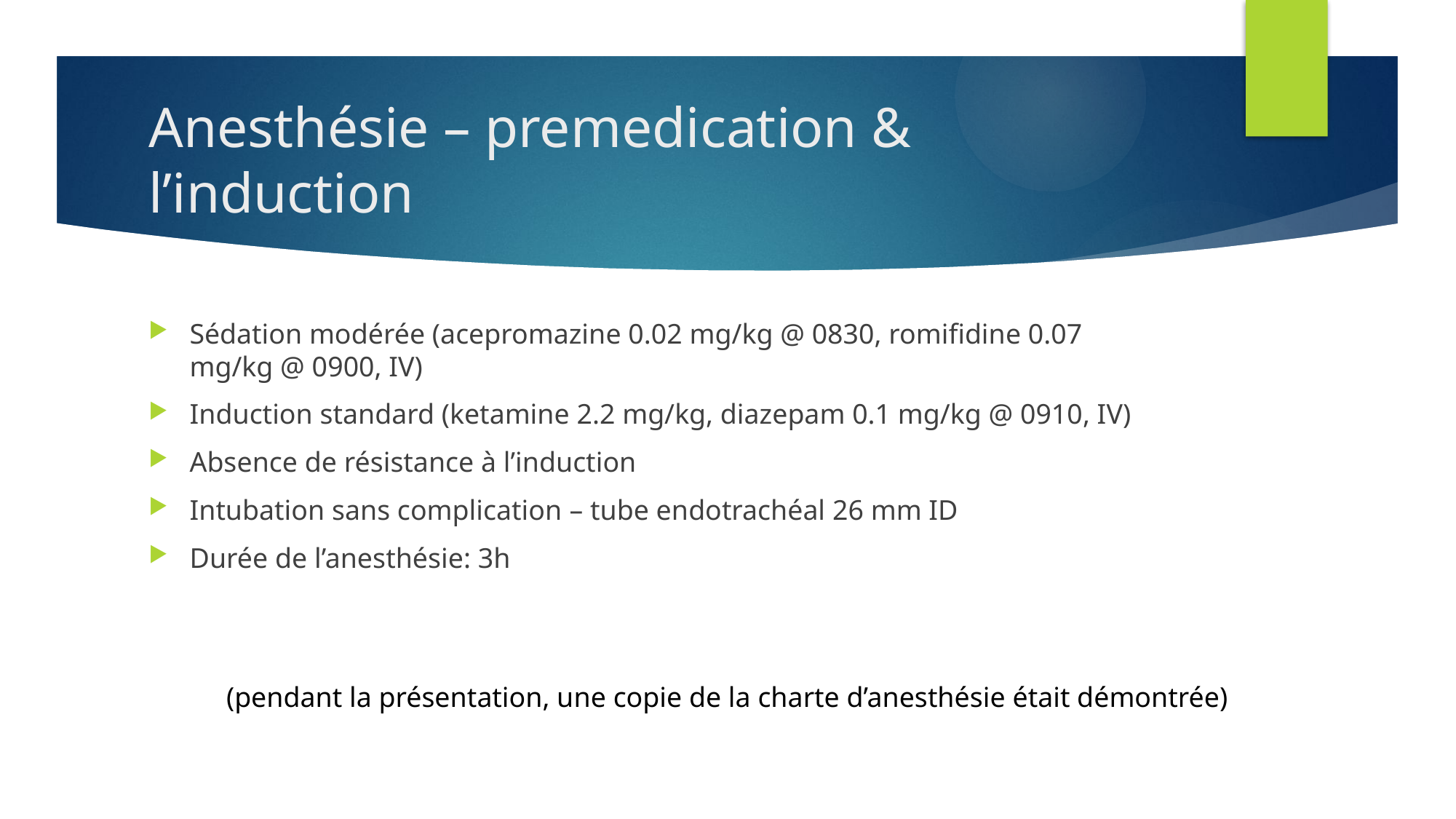

# Anesthésie – premedication & l’induction
Sédation modérée (acepromazine 0.02 mg/kg @ 0830, romifidine 0.07 mg/kg @ 0900, IV)
Induction standard (ketamine 2.2 mg/kg, diazepam 0.1 mg/kg @ 0910, IV)
Absence de résistance à l’induction
Intubation sans complication – tube endotrachéal 26 mm ID
Durée de l’anesthésie: 3h
(pendant la présentation, une copie de la charte d’anesthésie était démontrée)

## Slide 7
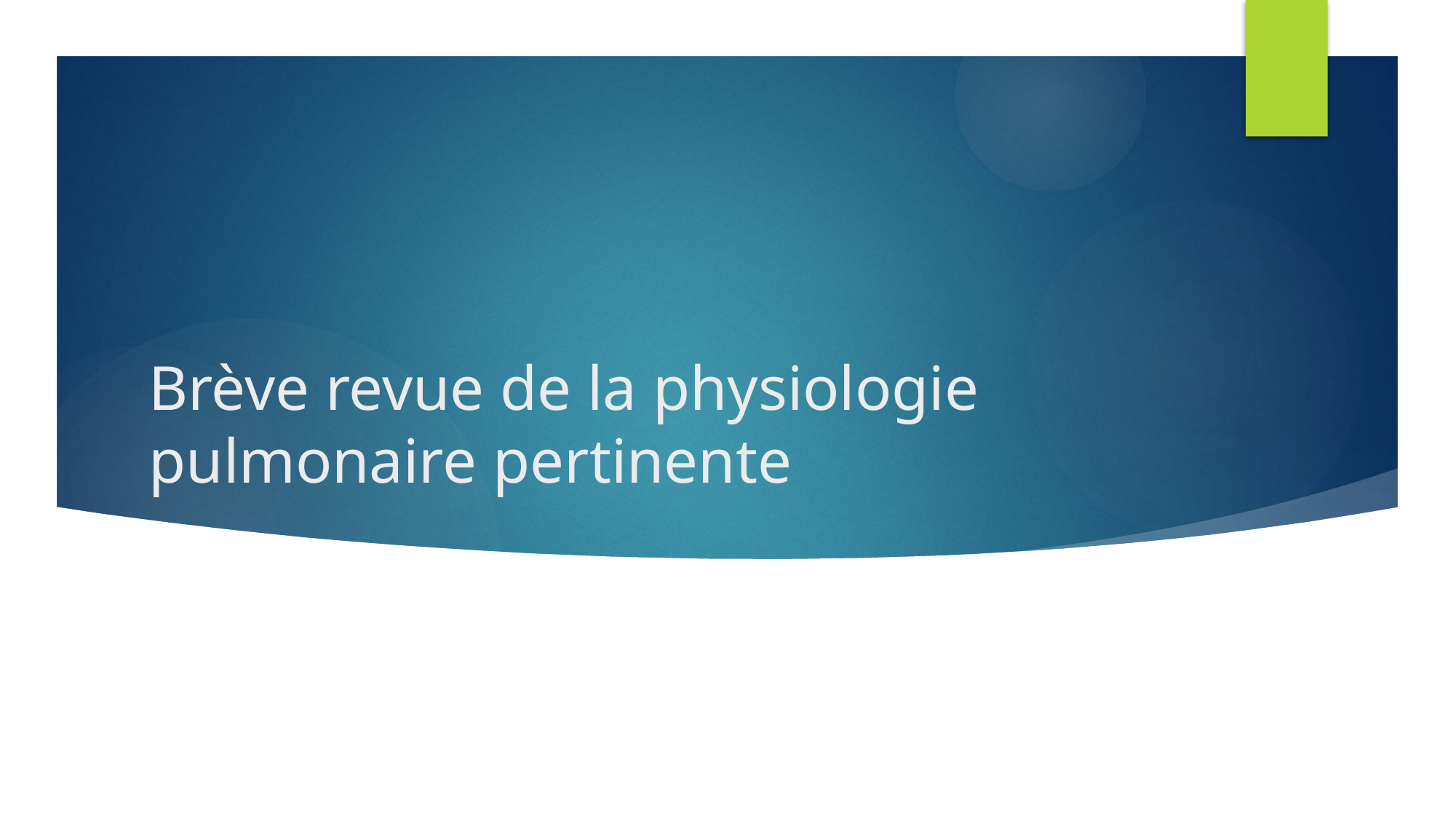

# Brève revue de la physiologie pulmonaire pertinente

## Slide 8
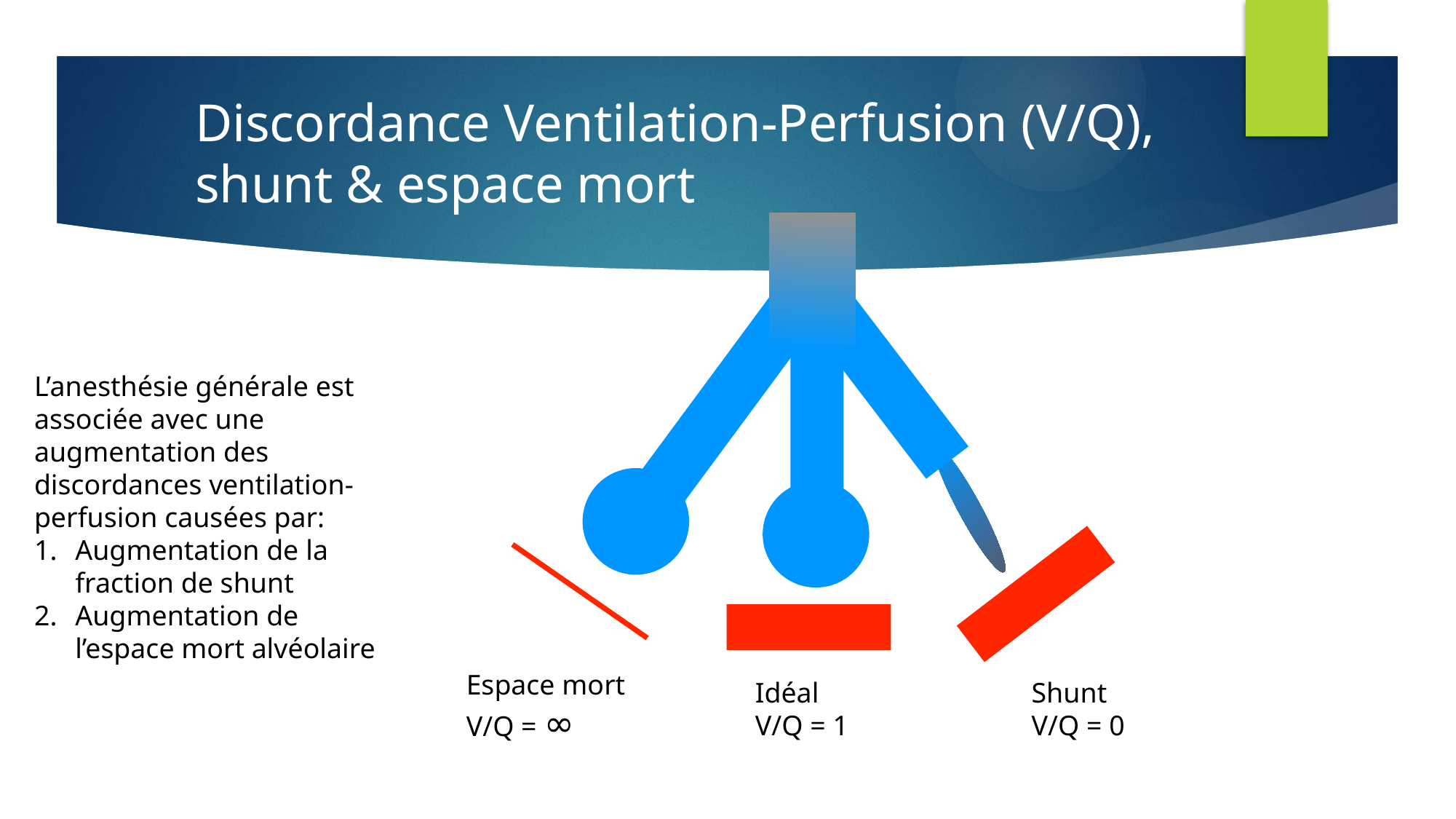

Discordance Ventilation-Perfusion (V/Q), shunt & espace mort
L’anesthésie générale est associée avec une augmentation des discordances ventilation-perfusion causées par:
Augmentation de la fraction de shunt
Augmentation de l’espace mort alvéolaire
Espace mort
V/Q = ∞
Idéal
V/Q = 1
Shunt
V/Q = 0

## Slide 9
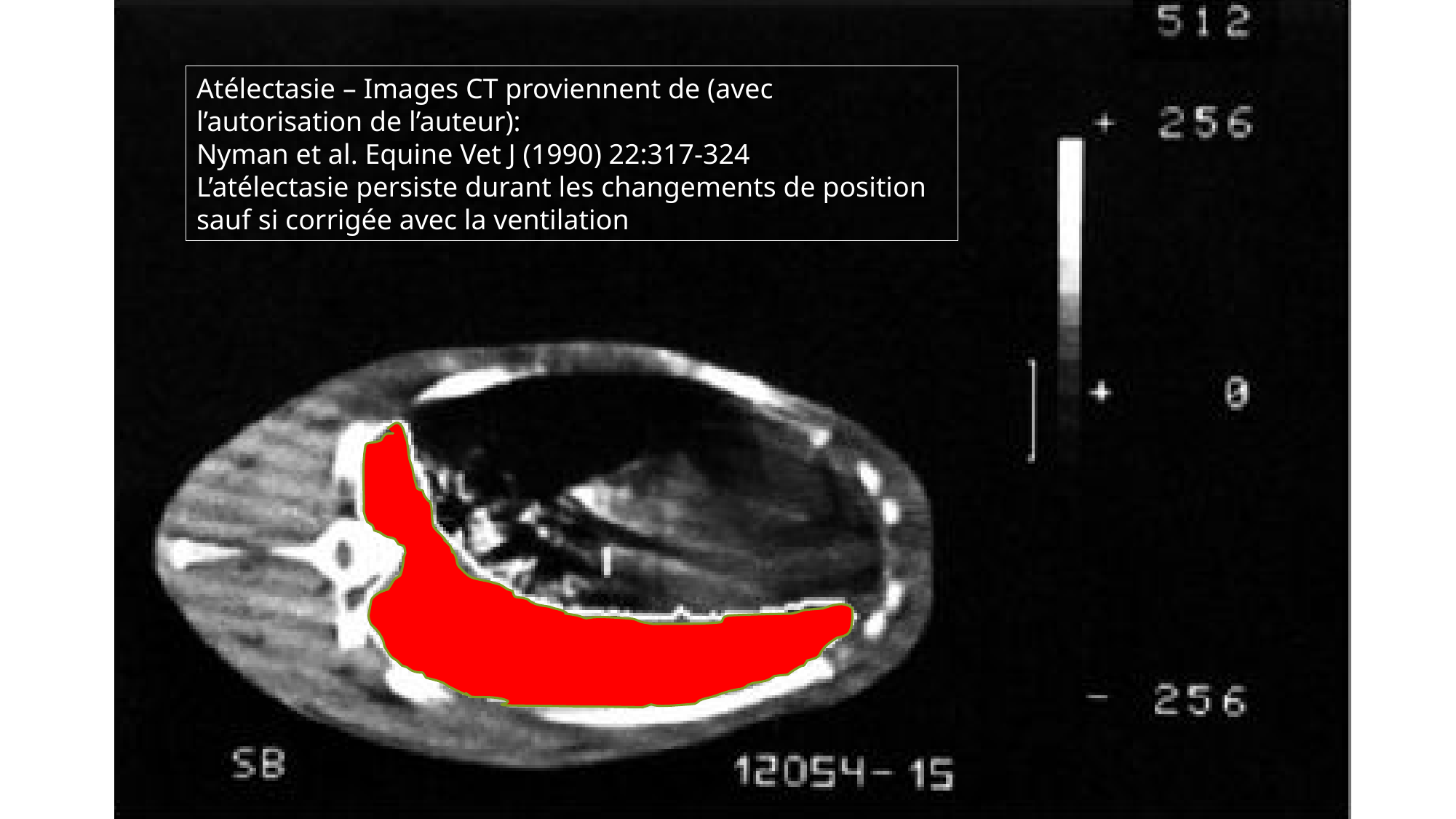

Atélectasie – Images CT proviennent de (avec l’autorisation de l’auteur):
Nyman et al. Equine Vet J (1990) 22:317-324
L’atélectasie persiste durant les changements de position sauf si corrigée avec la ventilation

## Slide 10
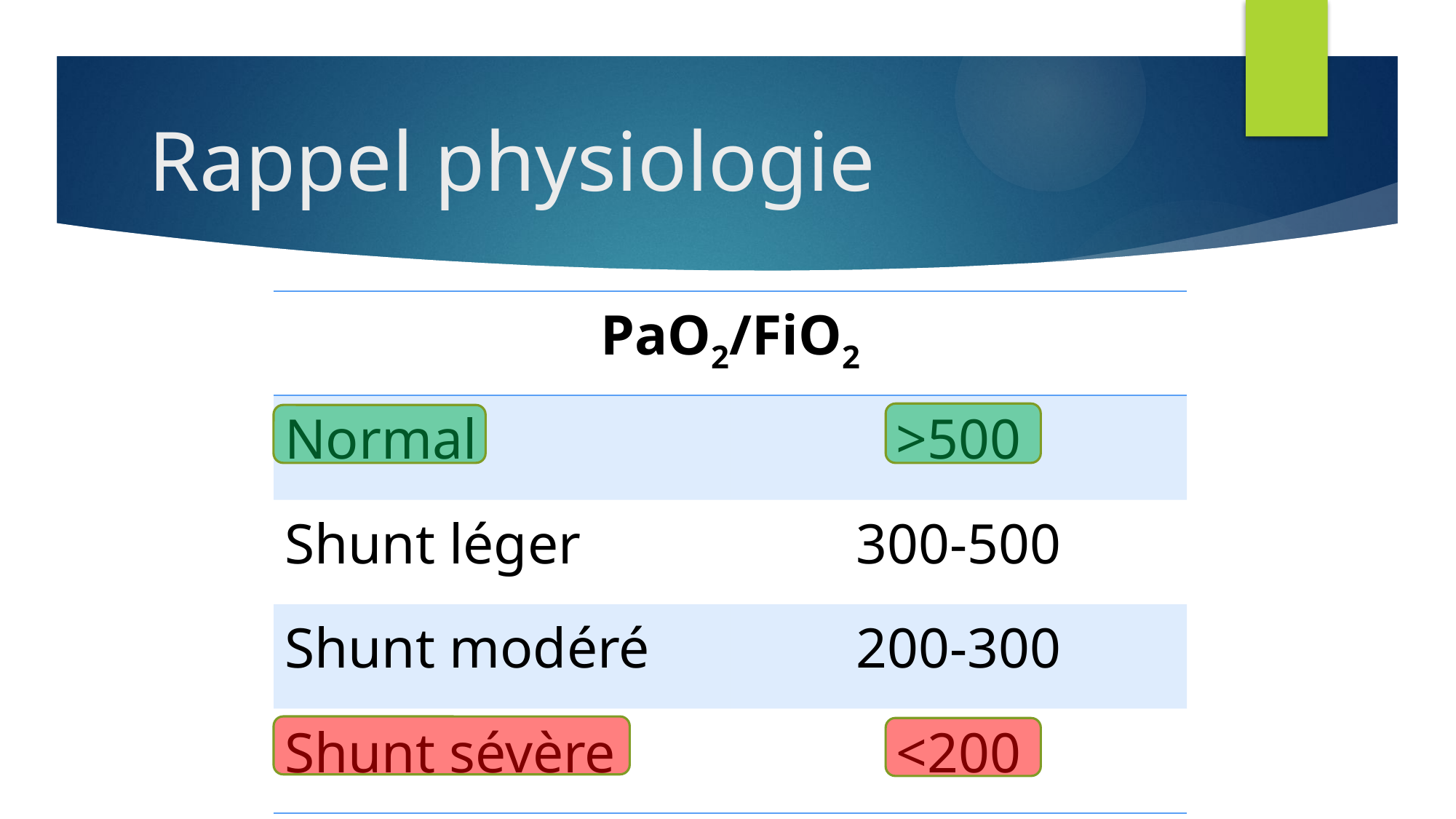

# Rappel physiologie
| PaO2/FiO2 | |
| --- | --- |
| Normal | >500 |
| Shunt léger | 300-500 |
| Shunt modéré | 200-300 |
| Shunt sévère | <200 |

## Slide 11
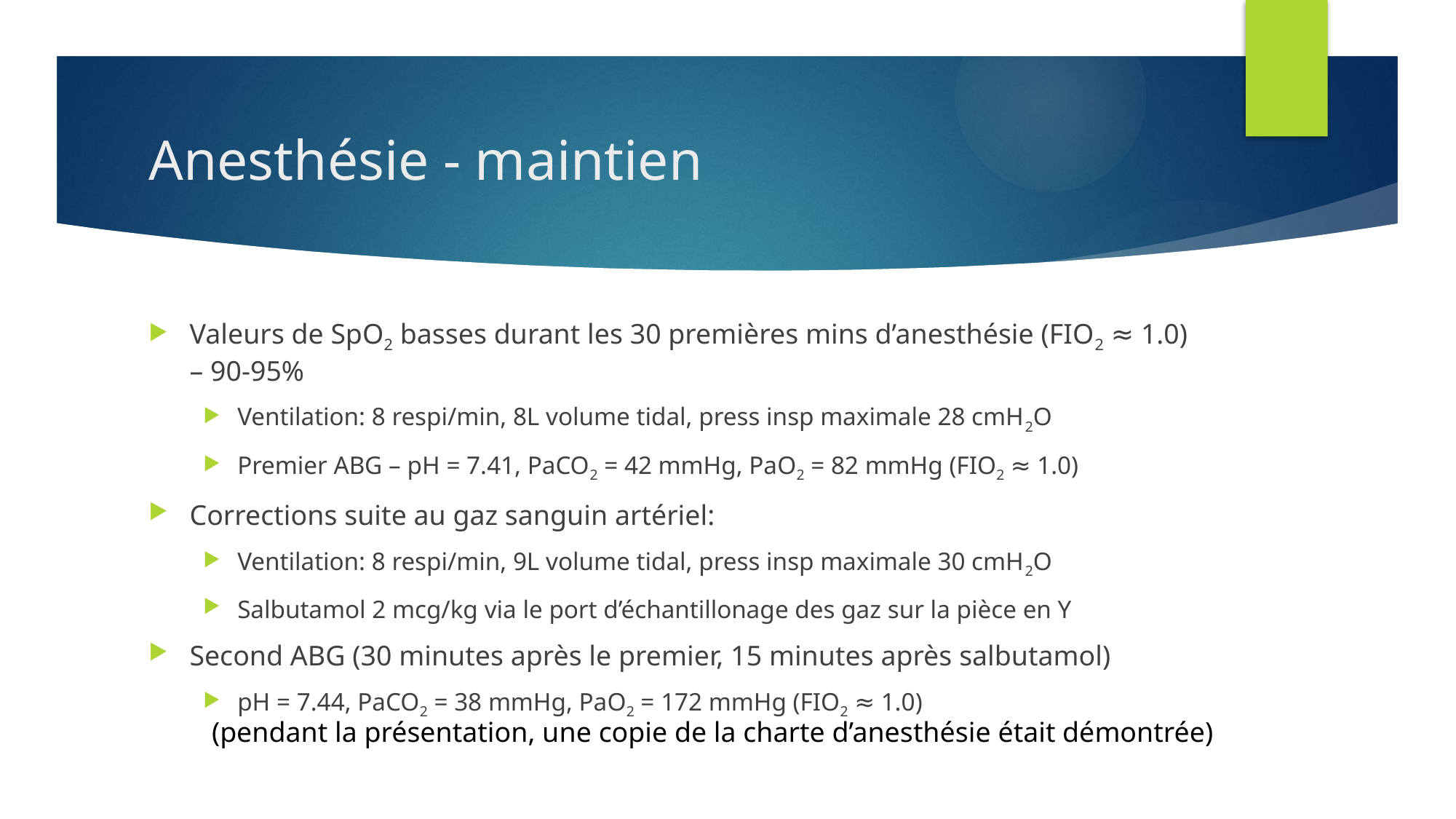

# Anesthésie - maintien
Valeurs de SpO2 basses durant les 30 premières mins d’anesthésie (FIO2 ≈ 1.0) – 90-95%
Ventilation: 8 respi/min, 8L volume tidal, press insp maximale 28 cmH2O
Premier ABG – pH = 7.41, PaCO2 = 42 mmHg, PaO2 = 82 mmHg (FIO2 ≈ 1.0)
Corrections suite au gaz sanguin artériel:
Ventilation: 8 respi/min, 9L volume tidal, press insp maximale 30 cmH2O
Salbutamol 2 mcg/kg via le port d’échantillonage des gaz sur la pièce en Y
Second ABG (30 minutes après le premier, 15 minutes après salbutamol)
pH = 7.44, PaCO2 = 38 mmHg, PaO2 = 172 mmHg (FIO2 ≈ 1.0)
(pendant la présentation, une copie de la charte d’anesthésie était démontrée)

## Slide 12
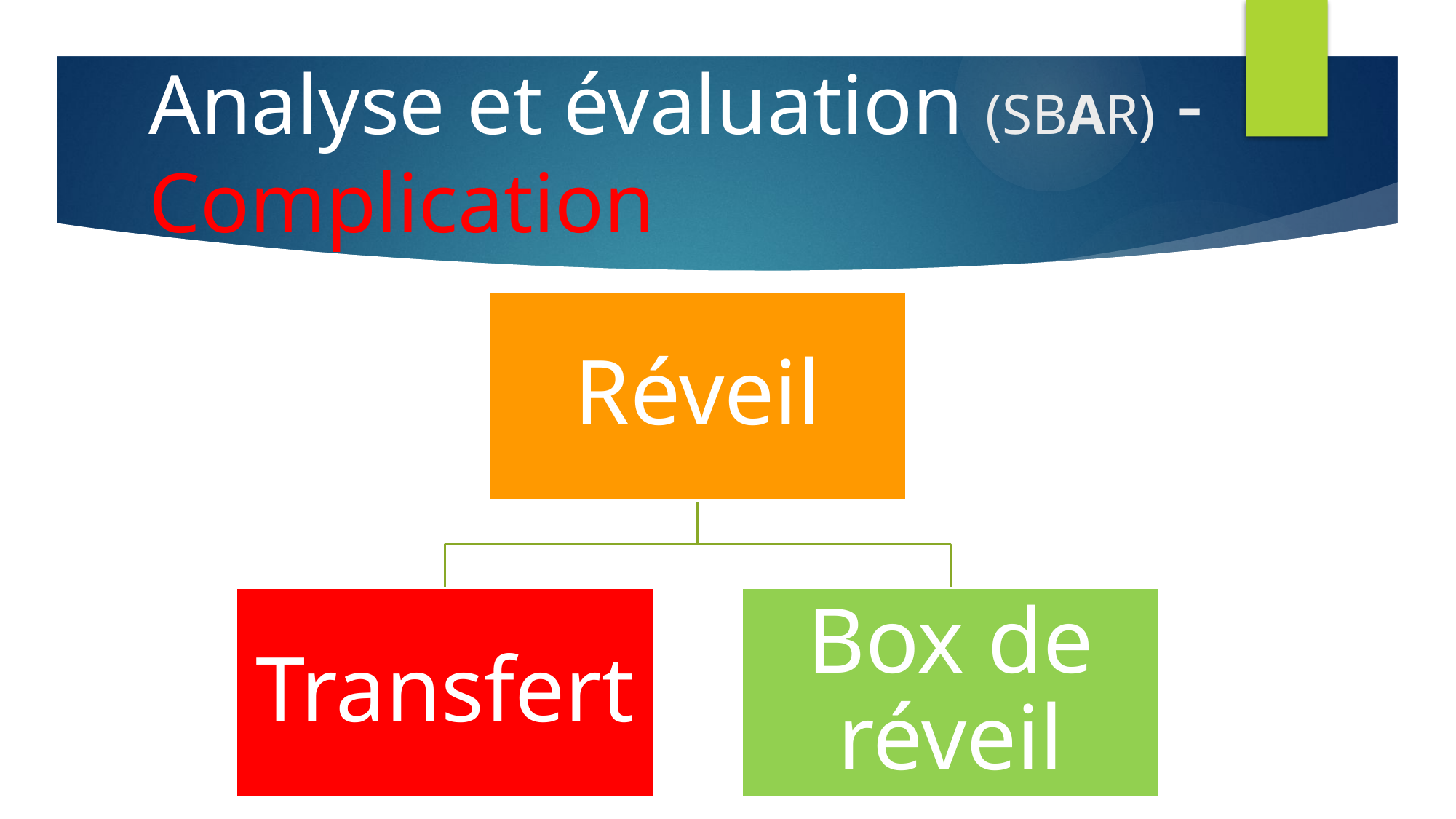

Analyse et évaluation (SBAR) - Complication

## Slide 13
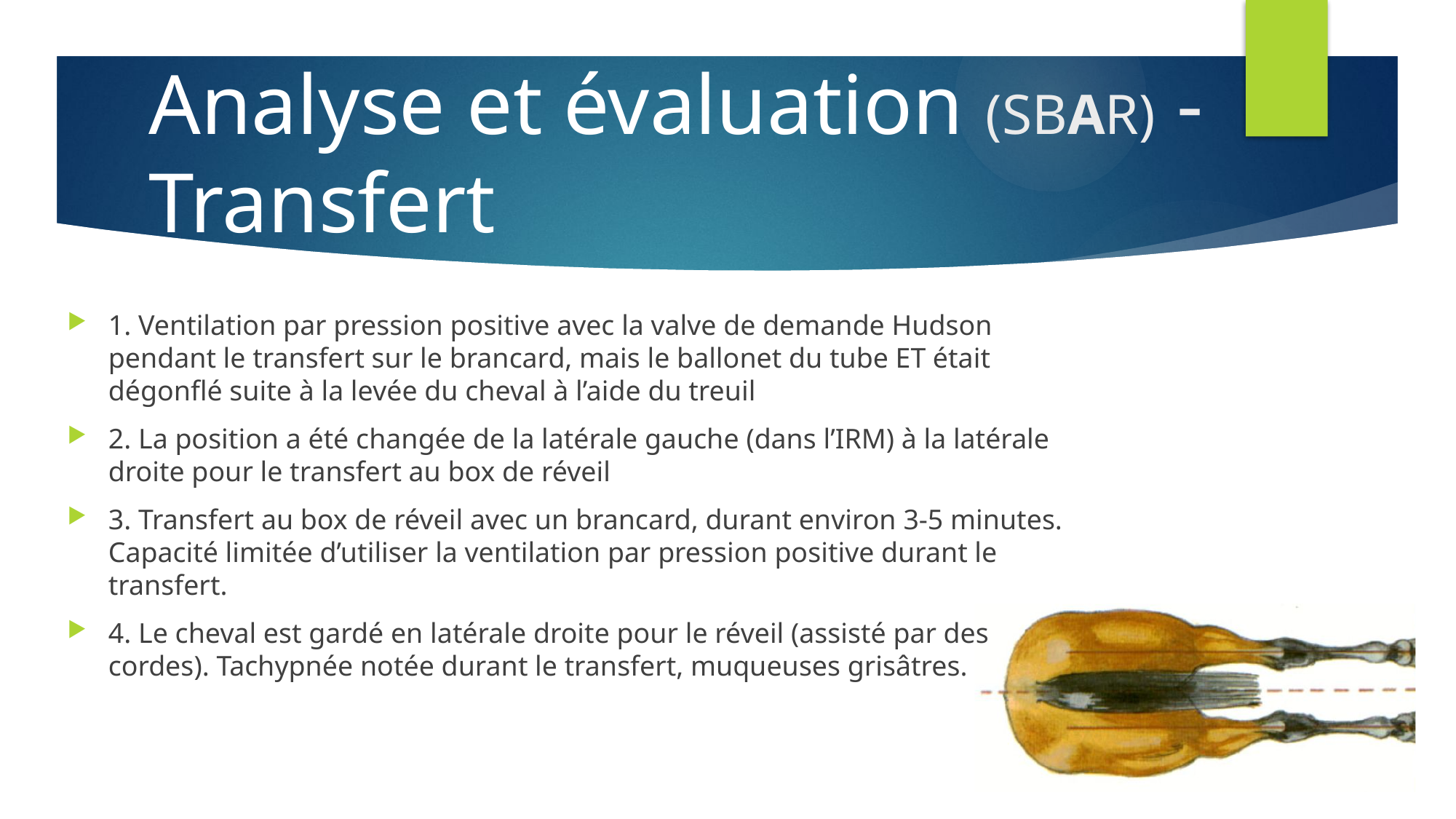

Analyse et évaluation (SBAR) - Transfert
1. Ventilation par pression positive avec la valve de demande Hudson pendant le transfert sur le brancard, mais le ballonet du tube ET était dégonflé suite à la levée du cheval à l’aide du treuil
2. La position a été changée de la latérale gauche (dans l’IRM) à la latérale droite pour le transfert au box de réveil
3. Transfert au box de réveil avec un brancard, durant environ 3-5 minutes. Capacité limitée d’utiliser la ventilation par pression positive durant le transfert.
4. Le cheval est gardé en latérale droite pour le réveil (assisté par des cordes). Tachypnée notée durant le transfert, muqueuses grisâtres.

## Slide 14
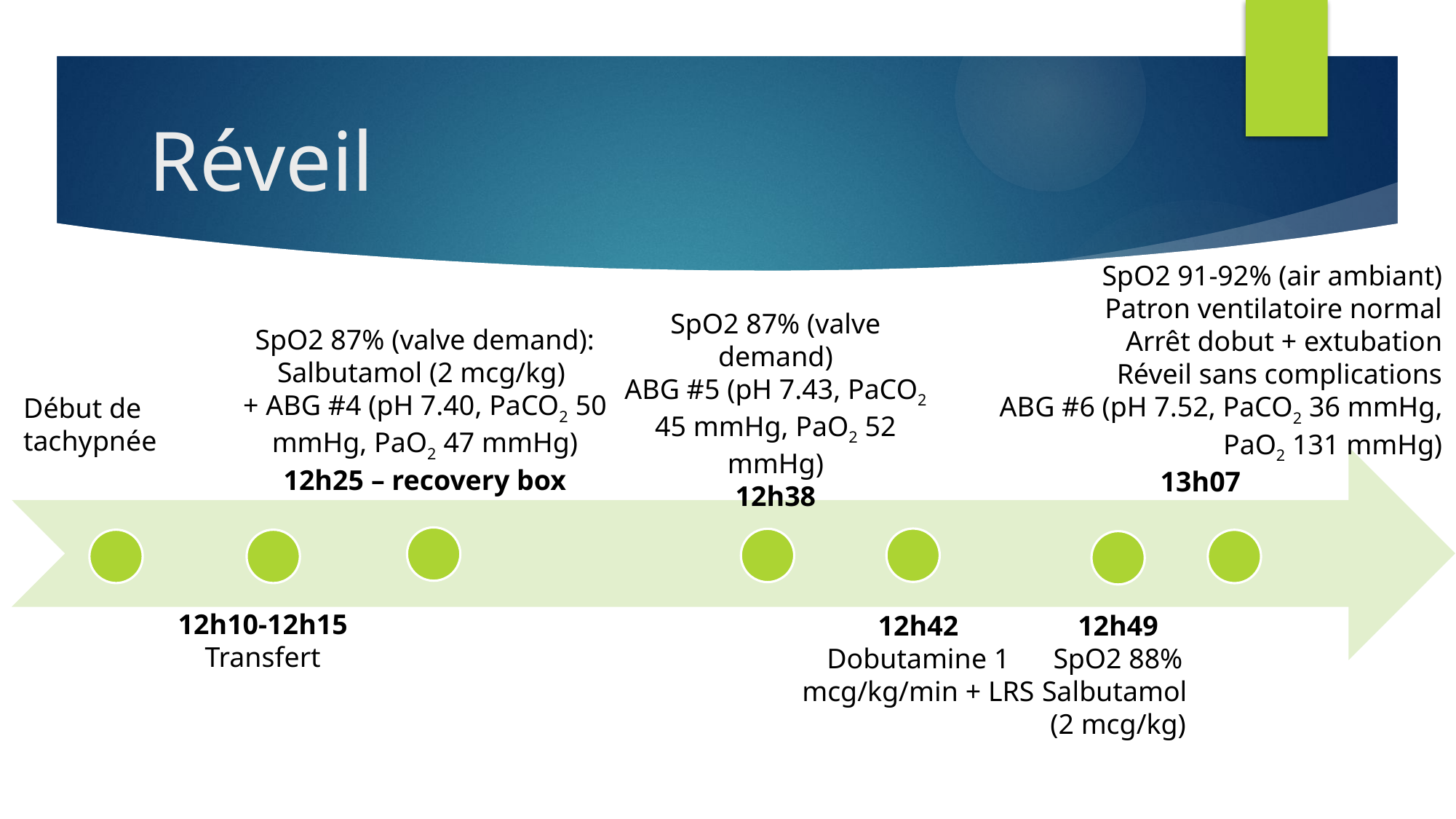

# Réveil
SpO2 91-92% (air ambiant)
Patron ventilatoire normal
Arrêt dobut + extubation
Réveil sans complications
ABG #6 (pH 7.52, PaCO2 36 mmHg, PaO2 131 mmHg)
13h07
SpO2 87% (valve demand)
ABG #5 (pH 7.43, PaCO2 45 mmHg, PaO2 52 mmHg)
12h38
SpO2 87% (valve demand):
Salbutamol (2 mcg/kg)
+ ABG #4 (pH 7.40, PaCO2 50 mmHg, PaO2 47 mmHg)
12h25 – recovery box
Début de tachypnée
12h10-12h15
Transfert
12h42
Dobutamine 1 mcg/kg/min + LRS
12h49
SpO2 88%
Salbutamol
(2 mcg/kg)

## Slide 15
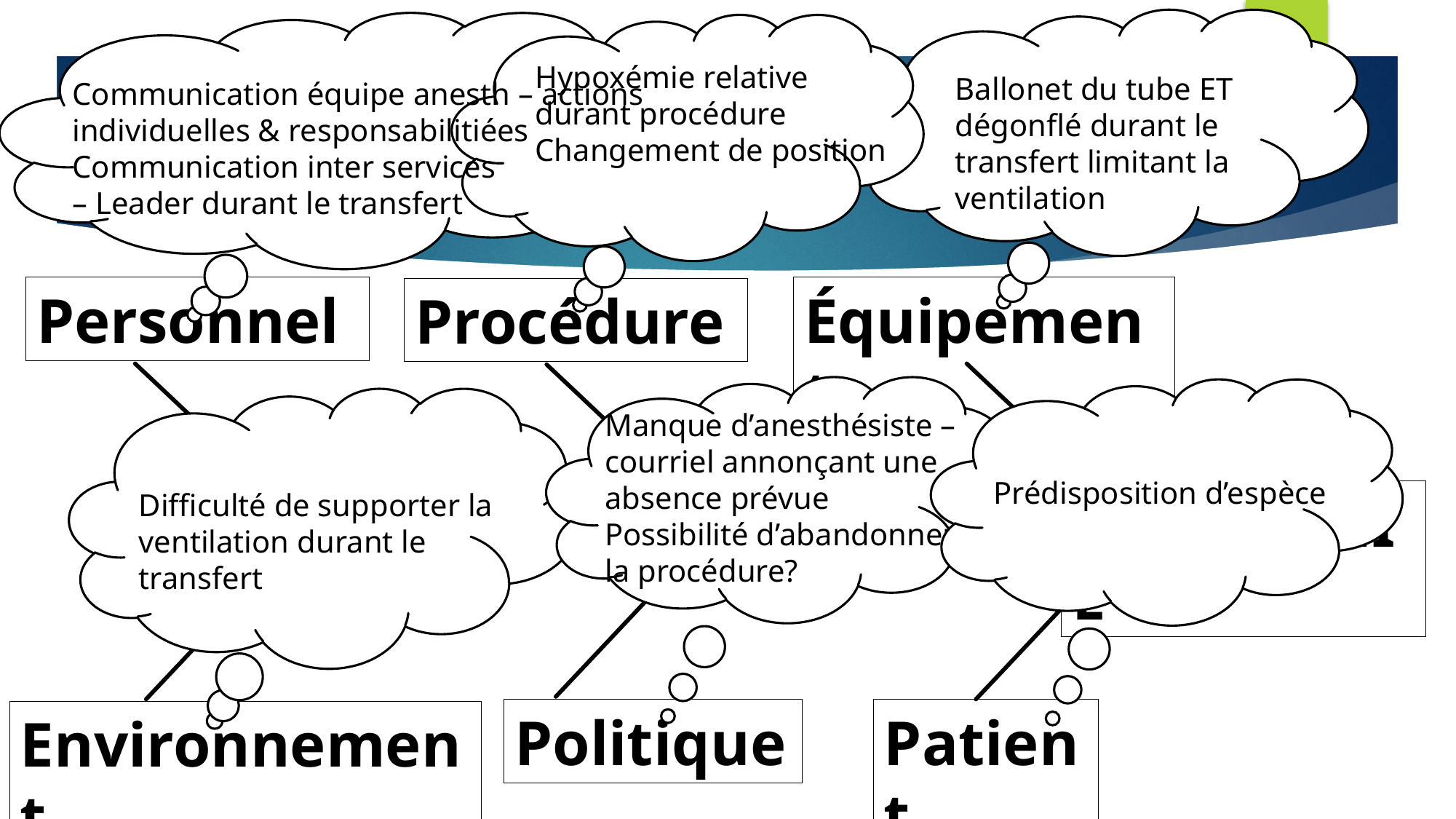

Hypoxémie relative durant procédure
Changement de position
Ballonet du tube ET dégonflé durant le transfert limitant la ventilation
Communication équipe anesth – actions individuelles & responsabilitiées
Communication inter services
– Leader durant le transfert
# Évaluation et analyse
Personnel
Équipement
Procédure
Manque d’anesthésiste – courriel annonçant une absence prévue
Possibilité d’abandonner la procédure?
Prédisposition d’espèce
Difficulté de supporter la ventilation durant le transfert
HYPOXÉMIE
Politique
Patient
Environnement

## Slide 16
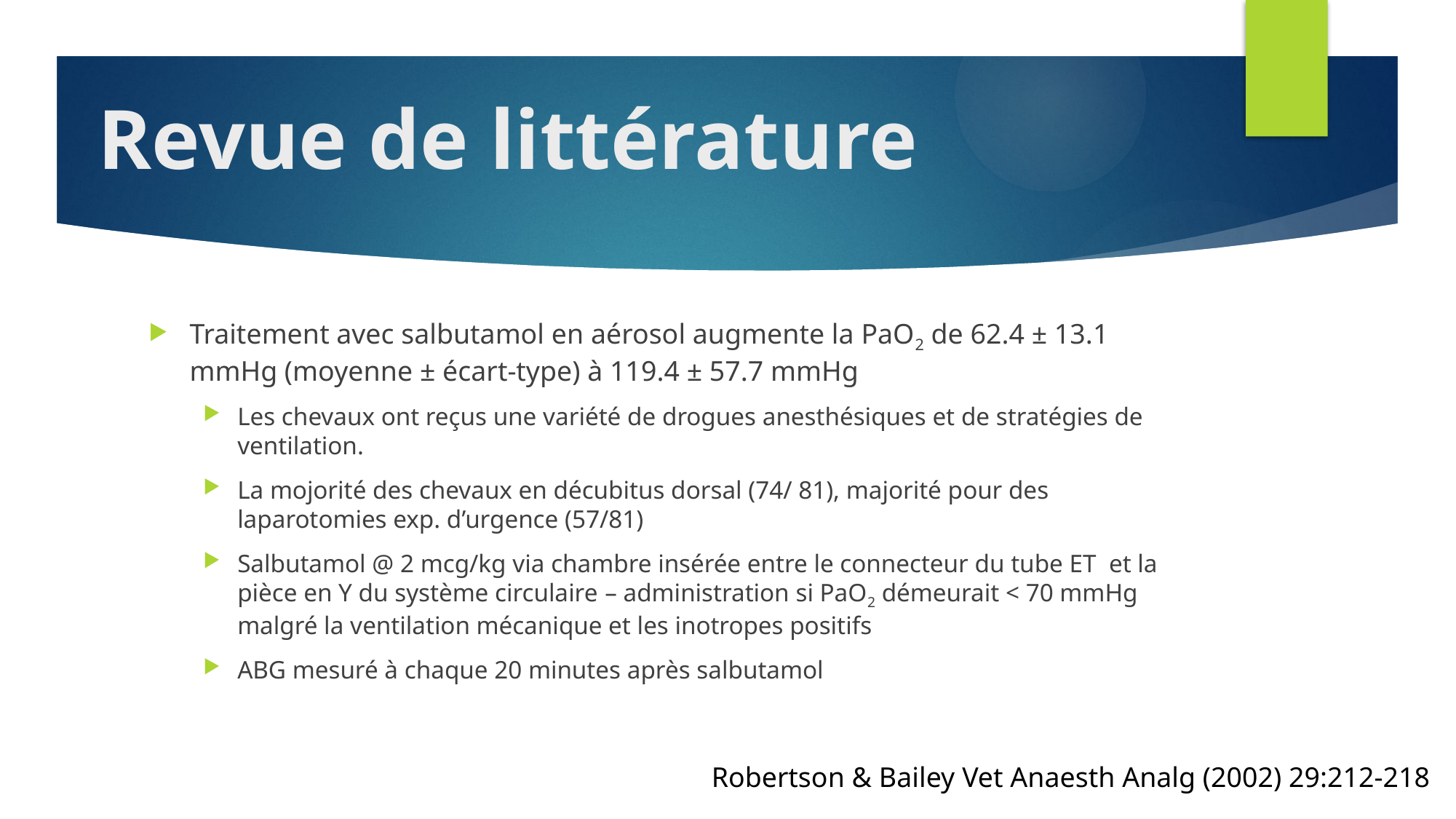

# Revue de littérature
Traitement avec salbutamol en aérosol augmente la PaO2 de 62.4 ± 13.1 mmHg (moyenne ± écart-type) à 119.4 ± 57.7 mmHg
Les chevaux ont reçus une variété de drogues anesthésiques et de stratégies de ventilation.
La mojorité des chevaux en décubitus dorsal (74/ 81), majorité pour des laparotomies exp. d’urgence (57/81)
Salbutamol @ 2 mcg/kg via chambre insérée entre le connecteur du tube ET et la pièce en Y du système circulaire – administration si PaO2 démeurait < 70 mmHg malgré la ventilation mécanique et les inotropes positifs
ABG mesuré à chaque 20 minutes après salbutamol
Robertson & Bailey Vet Anaesth Analg (2002) 29:212-218

## Slide 17
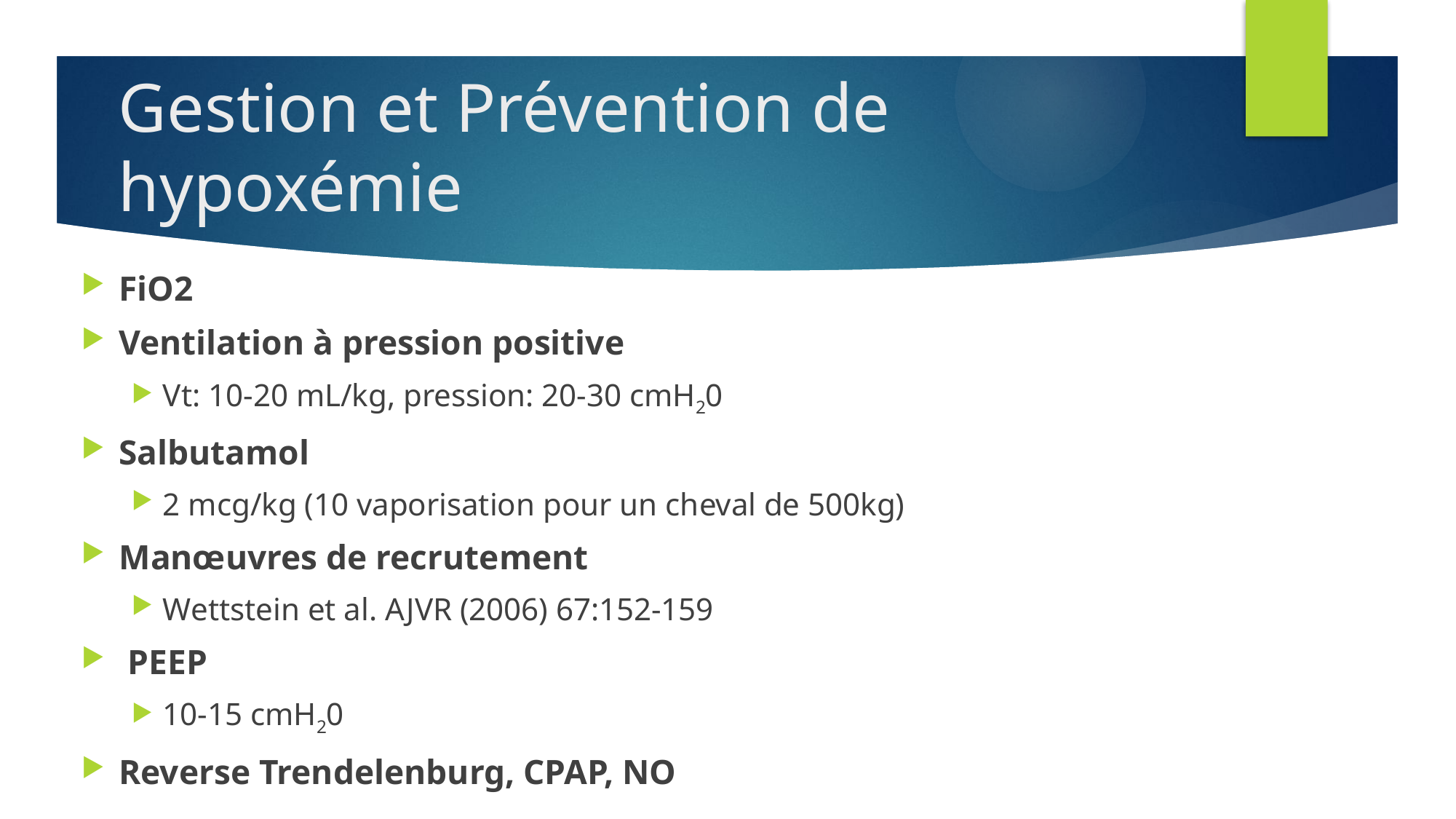

# Gestion et Prévention de hypoxémie
FiO2
Ventilation à pression positive
Vt: 10-20 mL/kg, pression: 20-30 cmH20
Salbutamol
2 mcg/kg (10 vaporisation pour un cheval de 500kg)
Manœuvres de recrutement
Wettstein et al. AJVR (2006) 67:152-159
 PEEP
10-15 cmH20
Reverse Trendelenburg, CPAP, NO

## Slide 18
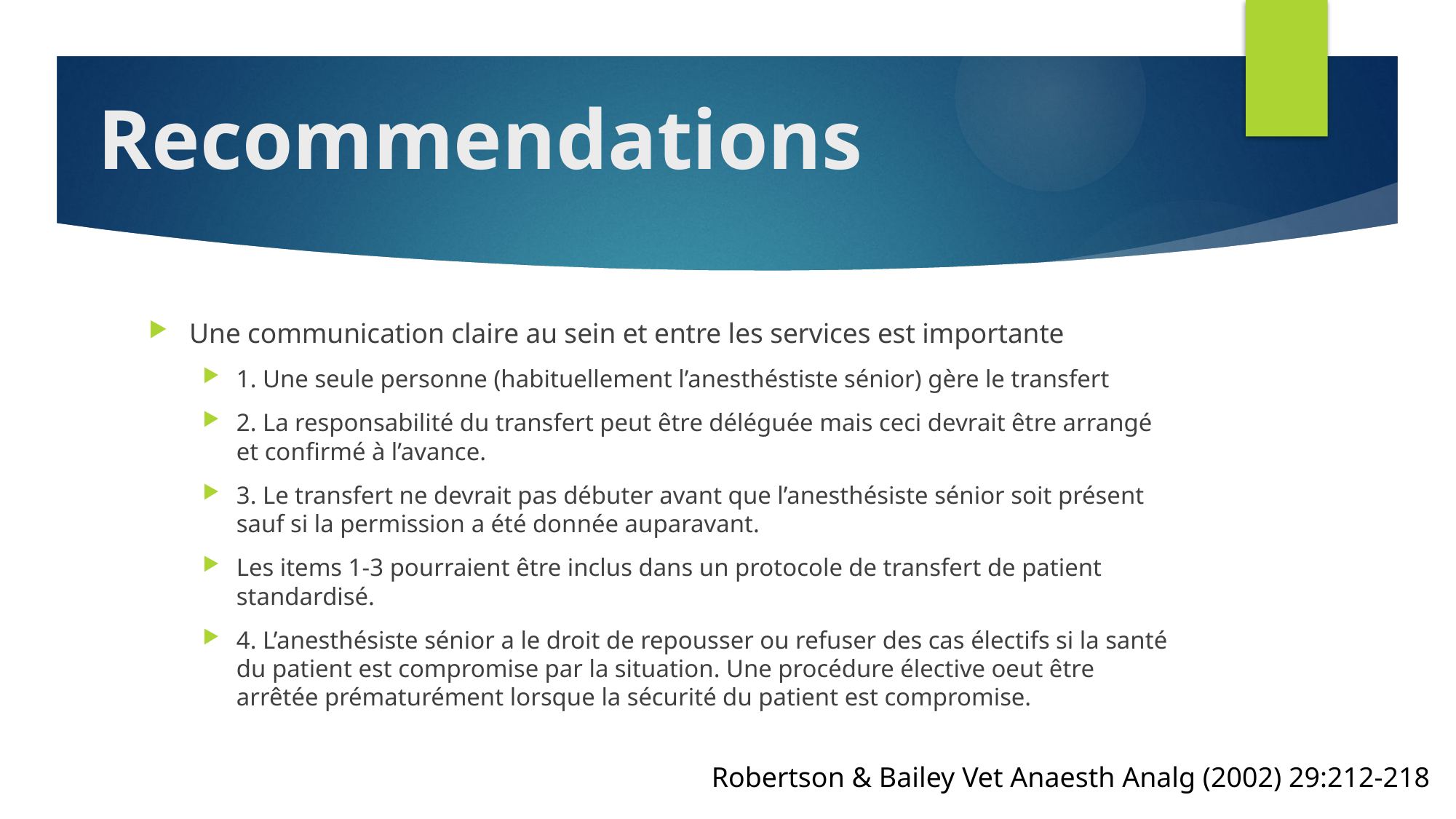

# Recommendations
Une communication claire au sein et entre les services est importante
1. Une seule personne (habituellement l’anesthéstiste sénior) gère le transfert
2. La responsabilité du transfert peut être déléguée mais ceci devrait être arrangé et confirmé à l’avance.
3. Le transfert ne devrait pas débuter avant que l’anesthésiste sénior soit présent sauf si la permission a été donnée auparavant.
Les items 1-3 pourraient être inclus dans un protocole de transfert de patient standardisé.
4. L’anesthésiste sénior a le droit de repousser ou refuser des cas électifs si la santé du patient est compromise par la situation. Une procédure élective oeut être arrêtée prématurément lorsque la sécurité du patient est compromise.
Robertson & Bailey Vet Anaesth Analg (2002) 29:212-218

## Slide 19
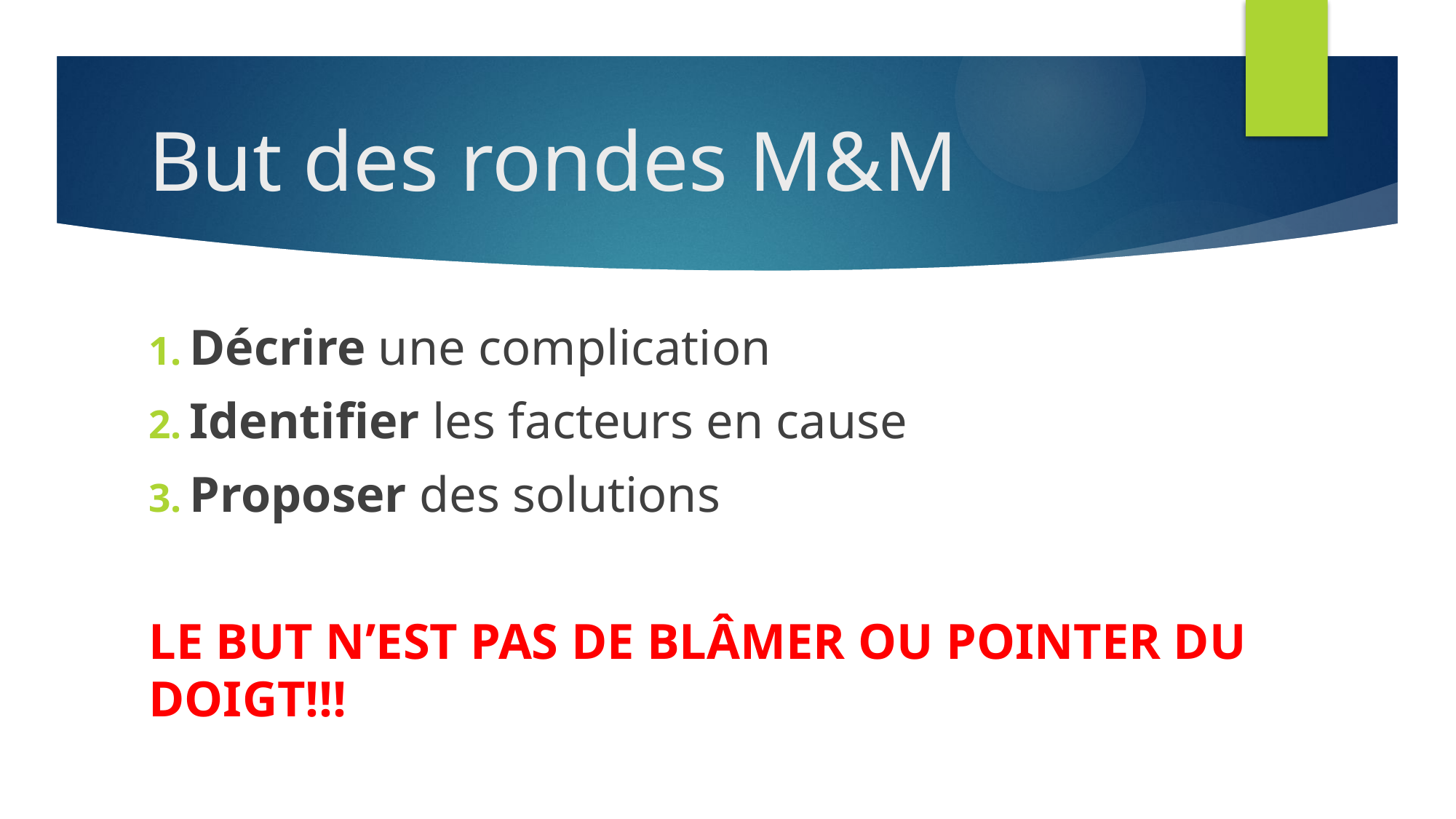

# But des rondes M&M
Décrire une complication
Identifier les facteurs en cause
Proposer des solutions
LE BUT N’EST PAS DE BLÂMER OU POINTER DU DOIGT!!!

## Slide 20
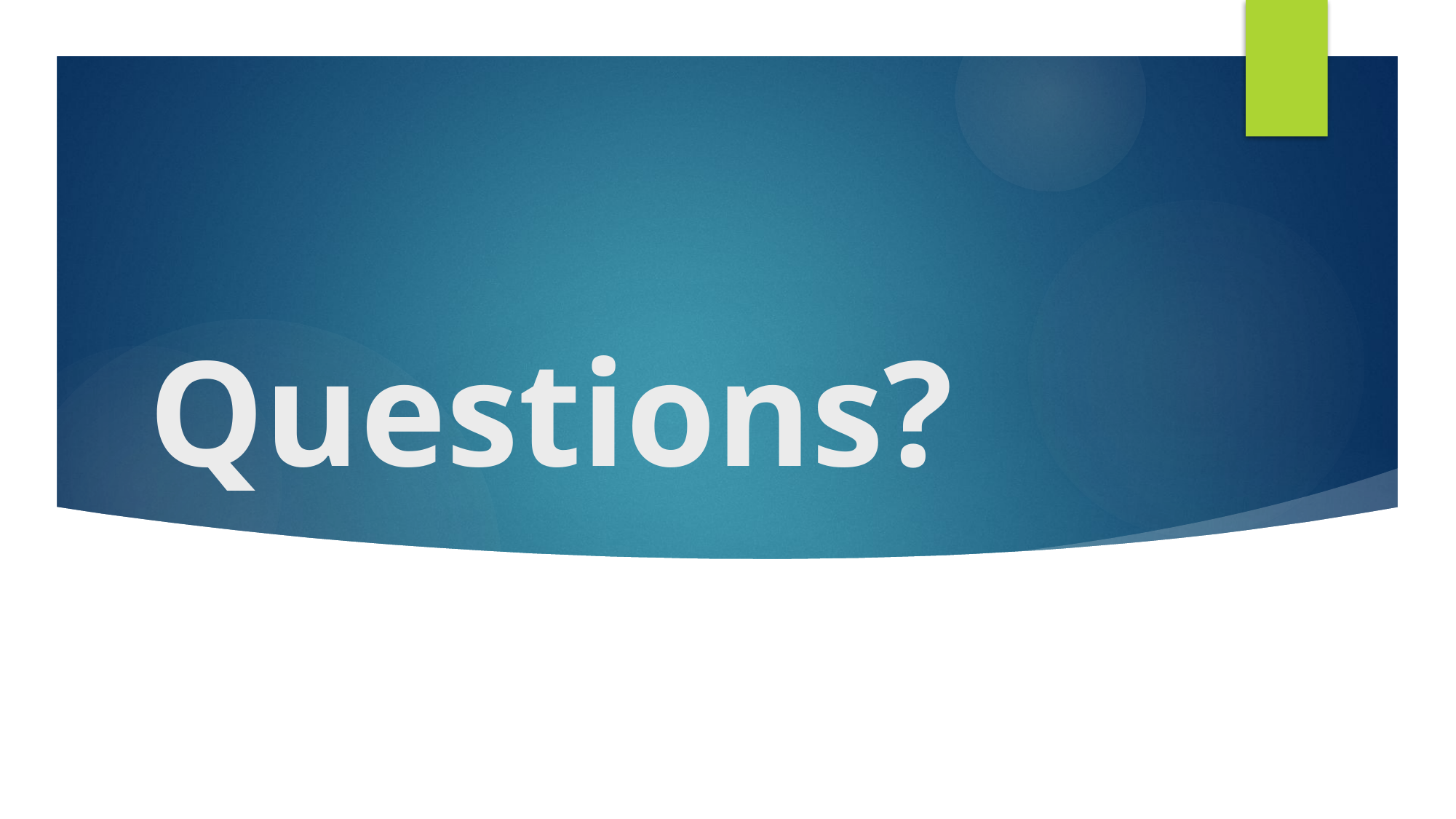

# Questions?
